# CTCF, BEAF-32, and CP190 are not required for the establishment of TADs in early *Drosophila* embryos but have locus-specific roles

**DOI:** 10.1126/sciadv.ade1085

**Published:** 2023-02-03

**Authors:** Gabriel R. Cavalheiro, Charles Girardot, Rebecca R. Viales, Tim Pollex, T. B. Ngoc Cao, Perrine Lacour, Songjie Feng, Adam Rabinowitz, Eileen E. M. Furlong

**Affiliations:** ^1^ European Molecular Biology Laboratory (EMBL), Genome Biology Unit, D-69117 Heidelberg, Baden-Württemberg, Germany.; ^2^ Collaboration for Joint PhD Degree between EMBL and Heidelberg University, Faculty of Biosciences, Baden-Württemberg, Germany.; ^3^ École Normale Supérieure, 45 rue d’Ulm, 75005 Paris, France.

## Abstract

The boundaries of topologically associating domains (TADs) are delimited by insulators and/or active promoters; however, how they are initially established during embryogenesis remains unclear. Here, we examined this during the first hours of *Drosophila* embryogenesis. DNA-FISH confirms that intra-TAD pairwise proximity is established during zygotic genome activation (ZGA) but with extensive cell-to-cell heterogeneity. Most newly formed boundaries are occupied by combinations of CTCF, BEAF-32, and/or CP190. Depleting each insulator individually from chromatin revealed that TADs can still establish, although with lower insulation, with a subset of boundaries (~10%) being more dependent on specific insulators. Some weakened boundaries have aberrant gene expression due to unconstrained enhancer activity. However, the majority of misexpressed genes have no obvious direct relationship to changes in domain-boundary insulation. Deletion of an active promoter (thereby blocking transcription) at one boundary had a greater impact than deleting the insulator-bound region itself. This suggests that cross-talk between insulators and active promoters and/or transcription might reinforce domain boundary insulation during embryogenesis.

## Teaser

Different boundaries require different subsets of insulator proteins, and in some cases an active promoter, for full insulation.

## INTRODUCTION

The organization of chromosomes into self-interacting chromatin domains, commonly called topologically associating domains (TADs), is a widespread feature in the animal kingdom (*1*–*4*). TADs are computationally defined from chromosome conformation capture data as genomic regions with enriched chromatin interaction frequencies when averaged across a population of cells (*2*, *4*–*6*). Enhancers and their target genes are often contained within the same TAD (*3*, *7*), although this organization varies from cell to cell (*5*). At some loci, domain boundaries insulate regulatory elements (enhancers and promoters) within the TAD from regulatory elements in neighboring domains, thus delimiting the domain in which enhancers drive transcriptional activation (*8*–*11*). However, at other loci, this does not appear to be the case as removal of the boundary or rearrangement of the TAD has little apparent impact on gene expression (*8*, *10*, *12*).

Most domain boundaries in mouse and human cells are occupied by the CCCTC-binding factor (CTCF), which binds to motifs in a convergent orientation at the two boundaries (*13*, *14*). Mechanistically, TADs are thought to form by a loop-extrusion mechanism in vertebrates, whereby a chromatin loop is extruded by the cohesin complex until it stalls at CTCF-bound regions (*15*, *16*). Depletion of CTCF in mouse embryonic stem cells or differentiated tissues reduced almost all (~80%) domain structure (*17*). Collectively, these studies demonstrate that CTCF is essential for the maintenance of the majority of TADs in vertebrates. However, it remains unclear how these domains are initially established during embryogenesis. Their formation coincides with zygotic genome activation (ZGA) or just after in zebrafish (*18*), mice (*19*, *20*), and *Drosophila* (*21*).

In addition to CTCF, *Drosophila* has a number of other insulator proteins (*22*). Of the direct DNA binding factors, CTCF, BEAF-32, and Su(Hw) bind to the majority of domain boundaries along with the architectural cofactors CP190 or Mod(Mdg4) (*4*, *23*–*29*). Many insulator proteins, including BEAF-32 (*30*) and CP190 (*31*), also bind very close to gene promoters, and a number of these factors have been proposed to function as transcriptional regulators (*30*–*33*). *Drosophila* boundaries also typically overlap transcribing promoters (*4*, *34*), representing ~77% of boundaries in *Drosophila* Kc167 cells (*23*), and it is currently not clear whether the enrichment of insulator proteins at domain boundaries is required for boundary formation or secondary to their role in the transcription of genes located at boundaries. TADs, especially in *Drosophila*, also reflect chromatin state, which, in turn, reflects transcription, and partitioning of chromatin states between domains of histone acetylation and methylation (especially H3K27me3) has been proposed to lead to TAD formation in *Drosophila* (*35*–*37*). Actually, transcriptional state is sufficient to predict Hi-C domain structure in a number of species (*34*), including flies. However, the relative contribution of insulator protein binding, transcription, or a combination of both to the formation and/or maintenance of TADs during embryonic development remains unclear.

A number of studies have begun to address this by genetic deletion or depletion of different factors in trans. For example, a complete deletion of CTCF (removing both the maternal and zygotic supply) *in vivo*revealed that CTCF is not required for *Drosophila* embryogenesis (*38*) or for the maintenance of TAD structure in the larval nervous system (*29*), but it is required for boundary insulation and correct gene expression at a small subset of loci (*29*, *38*). Depletion of BEAF-32 in Kc167 cells also had little global impact on TAD structure (*23*), while depletion in BG-3 cells was reported to affect ~20% of strong boundaries (*39*). Depletion of both CP190 and Chromator together in BG-3 cells affected a subset of boundaries with repressive chromatin (H3K27me3), while it had little impact on active regions (*39*). Similarly, genetic deletion of CP190 affected insulation on the subset of *Drosophila* boundaries that do not contain active promoters [~23% in Kc167 cells (*23*)] and had little impact on others (*28*).

The current lack of global regulators required for the maintenance of TAD structure in studies that focused on late developmental stages or cell lines led us to speculate that insulator proteins are perhaps more relevant for the initial establishment of TAD boundaries in *Drosophila* rather than for their maintenance. Here, we assessed the functional requirement for three major insulator proteins in the establishment of TADs in early *Drosophila* embryos. We first reassessed the precise timing of when intra-TAD interactions and high-frequency loops are first established in individual embryos by measuring pairwise distances within three domains in single cells using DNA fluorescence in situ hybridization (FISH). Although the genes within these loci are expressed at different stages of embryogenesis, the pairwise interaction frequencies within the three TADs show similar temporal dynamics in their establishment across the very early stages of ZGA, spanning nuclear cycle (NC) 12 to 14. This not only confirms previous Hi-C measurements (*21*, *40*) but also revealed extensive cell-to-cell heterogeneity in these intra-TAD distances at NC14, in line with the extensive cell-to-cell variation in TADs previously observed at this stage (*41*). We then profiled the genome-wide binding of five of the main insulator proteins [BEAF-32, CTCF, Su(Hw), CP190, and GAGA factor (GAF)] in a narrow window during the major wave of ZGA. All five proteins bind extensively along the genome and are present in various combinations at TAD boundaries.

We depleted BEAF-32, CTCF, or CP190 individually, removing their maternal contribution, and confirmed that all three proteins are depleted from chromatin in NC14 embryos. Evaluating their requirement for the establishment of chromatin topology, using both Hi-C and DNA-FISH, revealed that the majority of TADs are still able to form in the absence of these proteins, although with lower insulation. Boundaries that are occupied by different combinations of insulator proteins are associated with different levels of insulation at NC14 in wild-type (WT) embryos. After insulator protein depletion, 6 to 10% of boundaries have different sensitivities to the loss of a single factor, suggesting that it is not simply redundancy between any combination of insulators. Examining the impact of insulator protein depletion on gene expression revealed that ZGA occurs largely unperturbed. A few hundred genes are misexpressed, which appears to arise through multiple mechanisms. A subset of down-regulated genes (~2 to 20%) are bound by insulator proteins at their promoter and could be regulated directly or through the disruption of enhancer-promoter looping, while 6 to 13% of the up-regulated genes may occur through enhancer hijacking at weakened TAD boundaries. However, the majority (>80%) of all misexpressed genes have no obvious relationship to changes in topology and may represent more secondary indirect effects. To disentangle the interplay between insulator proteins and transcription itself, we dissected one TAD boundary that contains an active promoter during ZGA and a bound insulator binding site. Depletion of the activator protein Zelda (Zld) *in trans* and genetic deletion of the regulatory elements *in cis* indicate that removal of the active promoter (and thereby transcription) had a greater impact on TAD structure than removal of the insulator-bound region itself. This suggests that an active promoter or transcription (or some other aspect of the transcription factor’s function at the promoter) may feedback and reinforce domain insulation as embryogenesis proceeds.

## RESULTS

### The establishment of intra-TAD proximity during ZGA

**Fig. 1.**
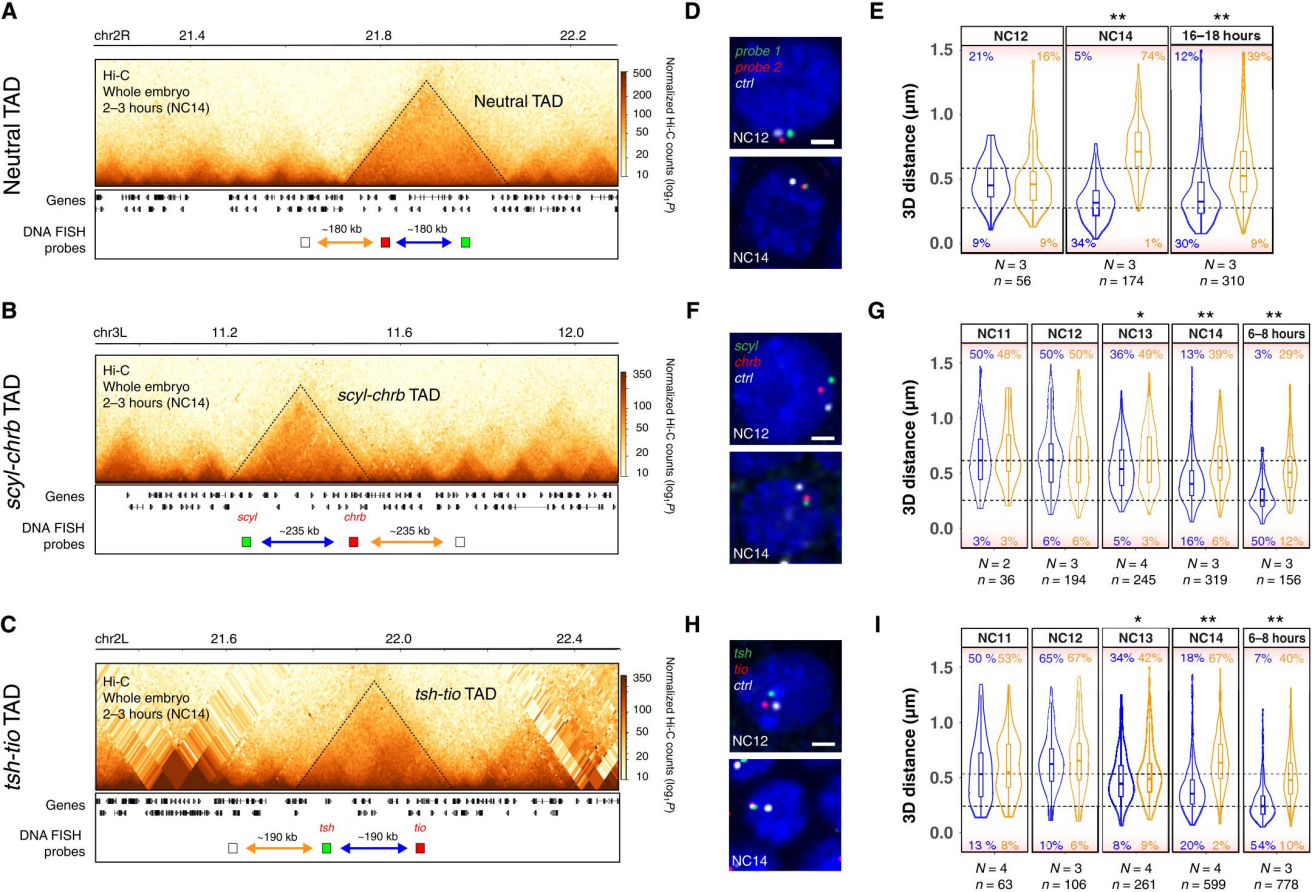
Intra-TAD proximity is established during ZGA, concomitant with TAD and loop formation. (**A** to **C**) Hi-C contact matrices from 2- to 3-hour WT embryos, showing a “neutral TAD” (A), *scyl-chrb* TAD (B), and *tsh-tio* TAD (C). Location of DNA-FISH probes indicated below (blue arrow, intra-TAD distance; orange arrow, inter-TAD distance). (**D**, **F**, and **H**) Representative images of DNA-FISH (single confocal Z section, ×100 magnification). The genomic regions targeted by FISH probes are indicated in (A) to (C) with the same colors (red, green, and white), with DAPI in blue. Scale bars, 1 μm. (**E**, **G**, and **I**) Quantification of 3D distances between the centers of mass of single spots corresponding to DNA-FISH probes in a given nucleus of embryos at each discrete stage (blue, intra-TAD point-to-point distance; orange, inter-TAD distance; *N*, number of embryos; *n*, number of alleles). Dotted lines correspond to 250-nm (bottom) and 600-nm (top) distances between probes. Percentages indicate the number of alleles with pairwise distances of >600 nm (top) or <250 nm (bottom). Kolmogorov-Smirnov test, *P < 0.01 and **P < 0.001.

TADs were initially detected by Hi-C in early *Drosophila* embryos starting at NC14, during the major wave of ZGA (*21*, *40*), which we confirmed here by performing Hi-C on tightly staged 2- to 3-hour embryos (predominantly NC14) (examples in Fig. 1, A to C). To more precisely quantify the timing of intra-TAD interactions, we performed DNA-FISH measuring pairwise distances within three TADs across different stages of the ZGA. One TAD has relatively uniform interaction frequencies throughout the domain, as measured by Hi-C (referred to as a neutral TAD; Fig. 1A), and contains genes that are not expressed during embryogenesis, with the exception of one gene (*CG9304*). The other two contain high-frequency loops between paralogous genes (*scyl*/*chrb* and *tsh*/*tio*; Fig. 1, B and C), which were shown to form at the *doc* locus even before the establishment of TADs (*41*). The DNA-FISH probes (~7 to 8.5 kb) were designed to target the outermost loop and an equidistant (in the linear genome) control probe outside the TAD (Fig. 1, A to C).

For the neutral TAD, at NC12 (the minor wave of ZGA), the pairwise distance between intra-TAD and inter-TAD probes is indistinguishable, with a small proportion of alleles (9%) showing overlap, defined as distances of <250 nm (Fig. 1, D and E, bottom dashed line). This indicates that in the vast majority of cells (>90%), the intra-TAD proximity of these regions has not yet formed. However, at NC14, the overlap between the intra-TAD probes greatly increased (34% of cells have pairwise distances of <250mn), concomitant with the major wave of ZGA, which was not observed for the inter-TAD probes (1% of alleles) (Fig. 1E). This proximity is maintained in later developmental stages (16 to 18 hours; Fig. 1E). Similarly, the high-frequency loops in the *scyl/chrb* and *tsh/tio* TADs are not present at the earlier stages (NC11 and NC12); the distances between the intra-TAD probes and the control probe are indistinguishable (Fig. 1, F to I). The formation of the loops initiates at NC13 (as observed by the increased proximity between the intra-TAD probes), extending a previous finding at the *doc* locus (*41*), and, to a much greater extent, at NC14: 16% of cells versus 6% have the loop anchor regions in close proximity (<250 nm) in the *scyl-chrb* TAD and 20% versus 2% in *tsh-tio* TAD (Fig. 1, G and I).

These single-cell measurements of intra-TAD distances also revealed extensive cell-to-cell heterogeneity within these TADs at NC14 (Fig. 1, E, G, and I). Although the pairwise intra-TAD proximity is higher at NC14 compared to that at NC11 and NC12, only 16 to 34% of cells (Fig. 1, E, G, and I) have pairwise proximity within 250 nm, indicating that there is still extensive cell-to-cell heterogeneity at NC14—for example, in 18% of cells, the outer-loop anchors within the *tsh/tio* TAD are at distances of >600 nm at NC14, indicating that the loop is not present in these cells at this stage (Fig. 1I, top dashed line). Similar extensive cell-to-cell heterogeneity was observed at the level of TADs during these stages (*41*, *42*). This cell-to-cell variability remained constant for the neutral TAD even at the end of embryogenesis (16 to 18 hours), where 30% of cells have distances of >250 nm (Fig. 1E). However, this changed quite markedly for the other two TADs where the number of cells with high proximity (<250 nm) between the loop anchors increased from 16 to 20% at NC14 to 50 to 54% of cells at mid-embryogenesis (6 to 8 hours, stage 11; Fig. 1, G and I). This may reflect the smaller size of nuclei at these later developmental stages, although this did not seem to affect intra-TAD pairwise distances within the neutral TAD. Alternatively, it may represent some reinforcement or stabilization of these high-frequency loops as embryogenesis proceeds. The cell-to-cell heterogeneity at NC14 also suggests that very defined TADs and intra-TAD proximity are not required for the regulation of gene expression during early stages of embryogenesis.

### Domain boundaries are occupied by diverse combinations of insulator proteins during the establishment of TADs

**Fig. 2.**
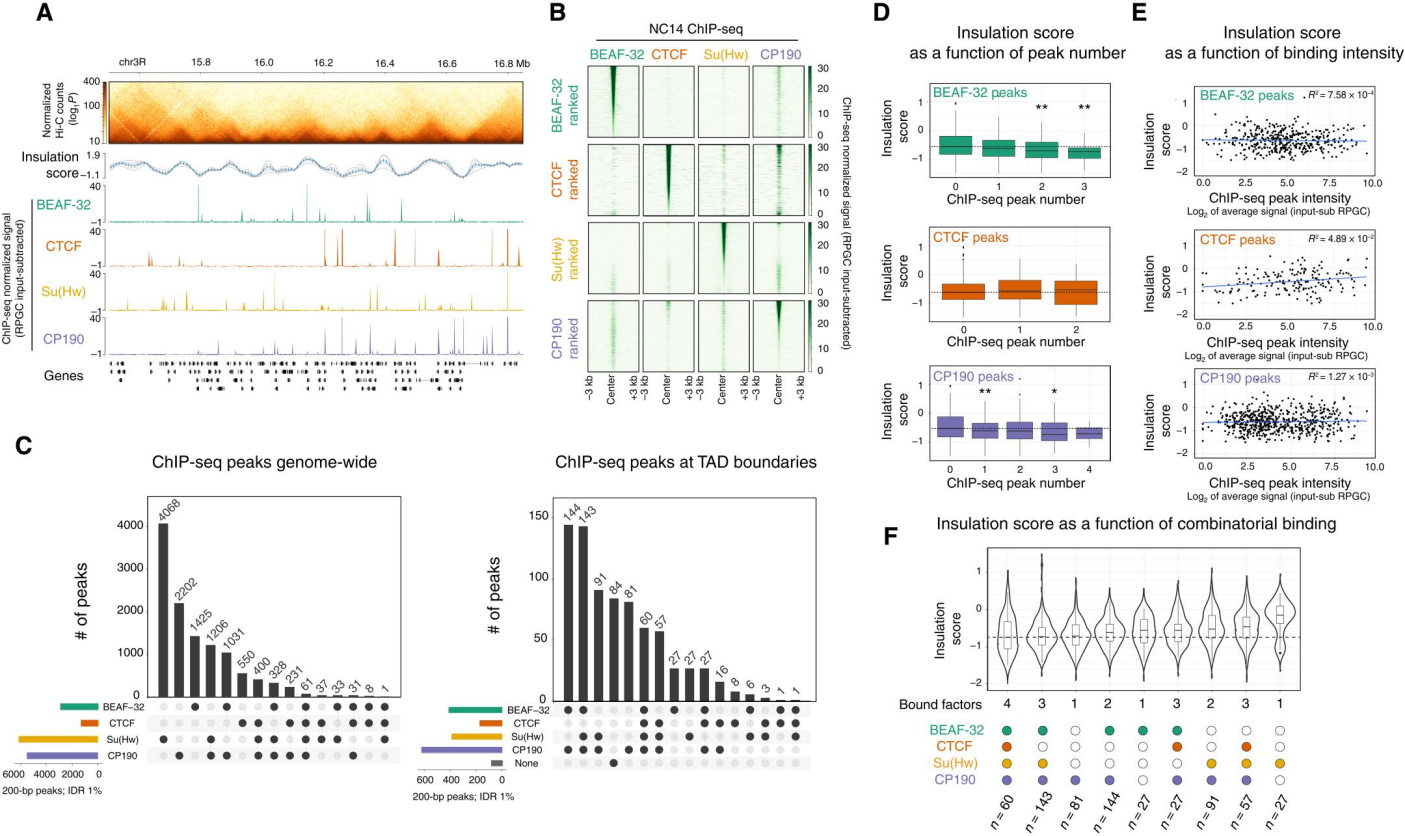
TAD boundaries are occupied by different combinations of insulator proteins during ZGA. (**A**) Hi-C matrix from 2- to 3-hour WT embryos (top), showing one genomic region with occupancy (ChIP-seq) of four insulator proteins at NC14. (**B**) Heatmap of insulator protein ChIP-seq normalized signal [reads per genome coverage (RPGC) input subtracted], centered at ChIP-seq peak summits. Each row is ranked by the ChIP-seq signal intensity of the indicated insulator protein, while the quantitative signal is shown for the other insulators. (**C**) UpSet plots showing the extent of insulator protein cobinding (summits within 200 bp genome-wide) (left) or at TAD boundaries (summits within the 10-kb boundary region) (right). (**D**) Box plots showing the distribution of insulation score at TAD boundaries (10-kb resolution) in 2- to 3-hour WT embryos as a function of the number of insulator ChIP peaks at the boundary. Kolmogorov-Smirnov, two-sided test, *P < 0.05 and **P < 0.01. (**E**) Scatterplots showing the distribution of insulation score at TAD boundaries in 2- to 3-hour WT embryos as a function of ChIP peak intensity (RPGC input subtracted) at the boundary. (**F**) Violin plots showing the distribution of insulation scores at TAD boundaries from 2- to 3-hour WT embryos as a function of insulator ChIP peaks at the boundary. Note that lower insulation score = higher insulation. Violin plots are ordered from the highest insulation (left) to the lowest (right).

To determine how TADs are initially established during ZGA, we assessed the requirement of insulator proteins given the role of CTCF in TAD formation in vertebrates, the occupancy of these proteins at TAD boundaries in *Drosophila* [shown for later embryonic stages and cell lines (*23*, *43*, *44*)], and the availability of these proteins in early embryos due to their maternal deposition. As the occupancy of these insulators had not been assessed at NC14, we first performed chromatin immunoprecipitation sequencing (ChIP-seq) on tightly staged NC14 embryos (2 hours and 10 min. to 2 hours and 40 min.) for the four most studied *Drosophila* insulator proteins BEAF-32, CTCF, Su(Hw), and CP190 (*22*, *43*) during NC14. Biological replicates for each factor are highly correlated (fig. S1, A and B), and the DNA binding motif for each insulator protein is highly enriched under their ChIP peaks (fig. S1C), attesting to the quality of these NC14 ChIP datasets. All four insulator proteins are significantly bound to thousands of genomic regions at NC14 (Fig. 2, A to C): BEAF-32, 2917; CTCF, 1319; Su(Hw), 6134; and CP190, 5490 peaks at <1% Irreproducible Discovery Rate (IDR) (Materials and Methods). Each insulator protein binds to many regions alone and in combinations with each other (Fig. 2, B and C), although the three insulators with direct DNA binding [BEAF-32, CTCF, and Su(Hw)] have different distributions. BEAF-32, CTCF, and CP190 combinatorial binding is more common: 51, 58, and 60% of their peaks colocalize with at least one other insulator protein, respectively, while this proportion was 34% for Su(Hw) (Fig. 2C, left). The most frequent combinations are [BEAF-32 & CP190] and [Su(Hw) & CP190], representing 35% of BEAF, 20% of Su(Hw), and 41% of CP190 peaks. Triple binding is rare in comparison to single or double and was always observed between CP190 and two other factors. Only 60 regions are cobound by all four factors, and all 60 of which are at TAD boundaries (Fig. 2C).

We also performed ChIP-seq on the transcription factor Trithorax-like (Trl/GAF) at NC14, which was recently proposed to bind to promoter-tethering elements to establish enhancer-promoter communication in *Drosophila* (*45*). Although GAF binds to TAD boundaries at NC14 (fig. S1E), we observed very little cobinding between GAF and the other insulator proteins examined, including CP190 (fig. S1, B and E).

CP190 is recruited to chromatin indirectly through protein-protein interactions with other insulator proteins and therefore does not have a canonical DNA binding motif (*46*–*48*). This is reflected in the extensive colocalization of CP190 with the other three insulator proteins (60% of peaks overlap; Fig. 2C). This is in keeping with the *de novo* motif enrichments under CP190 overlapping peaks, where Su(Hw), CTCF, and BEAF-32 motifs are within the first five most enriched motifs (fig. S1C). Motif analyses of 40% of CP190-only peaks identified additional putative recruiters of CP190 during these early stages of embryogenesis (fig. S1D), including the insulator protein Pita (*49*) and the transcriptional regulators Knirps, Jim, Nautilus, and Visual system homeobox 2.

We next assessed how insulator occupancy relates to the newly established TAD boundaries during ZGA. As the ability to define TAD boundaries in a given condition varies greatly depending on the resolution of the Hi-C matrices and the TAD calling algorithm used, we used our Hi-C data from tightly staged 2- to 3-hour embryos to call TADs at multiple base pair resolutions (2, 5, and 10 kb) and *q* values (0.1, 0.05, and 0.01) (fig. S2A). The 10-kb resolution and *q* value of 0.1 gave the most consistent results, showing a better visual overlap with TADs while avoiding splitting larger domains. By using these thresholds, we identified 772 high-confidence TAD boundaries in 2- to 3-hour embryos (Fig. 2A and fig. S2, A and B). Approximately 90% (703 of 772) of these domain boundaries have at least one of the four analyzed insulator proteins binding within the 10-kb boundary window at NC14 (Fig. 2C, right), with CP190 being the most frequent (80%, 624 of 772). TAD boundaries are preferentially bound by combinations of insulators rather than single proteins, with 70% of all boundaries being occupied by two or more of the four insulators (Fig. 2C, right). This proportion is likely an underestimate given that there are other *Drosophila* insulator proteins that we did not profile here. To assess that, we examined available ChIP data for Ibf1 (*50*), Ibf2 (*50*), Pita (*51*), ZIPIC (*51*), and Zw5 (*51*), which were performed in either cell lines or at later developmental stages (fig. S1F). Even assuming that the binding of these factors is the same at NC14, different combinations of BEAF-32, CTCF, Su(Hw), and CP190, without the other factors, cumulatively represent the largest binding classes at TAD boundaries at NC14. The most prevalent combinations (from our NC14 data) are [BEAF-32 & CP190] and [BEAF-32, Su(Hw), & CP190], present in 17 and 18% of TAD boundaries, respectively (Fig. 2C, right).

Two trends were previously reported in mice or *Drosophila* cell lines. In mammals, both a higher number and a higher intensity of CTCF ChIP peaks were proposed to provide robustness to TAD boundaries (*12*, *52*). Here, during the establishment of TADs in early *Drosophila* embryos, we observed a similar trend for stronger insulation at boundaries with multiple BEAF-32 or CP190 peaks, although this does not hold true for multiple CTCF peaks (note that a lower insulation score reflects higher boundary insulation; Fig. 2D). However, ChIP peak intensity at TAD boundaries for either BEAF-32, CTCF, or CP190 is not correlated with stronger insulation (Fig. 2E). In *Drosophila* Kc167 cells, the strength of TAD boundaries (as measured by insulation score) is correlated with the number of cobound insulator proteins (heterotypic binding) (*24*). We observe a similar trend in NC14 embryos: 26% (202 of 772) of domain boundaries are occupied by either all four (60 of 772) or three [BEAF-32, Su(Hw), and CP190] factors (Fig. 2C, right), and these are among those with the highest insulation (Fig. 2F). For boundaries occupied by other combinations of three or less insulator proteins, the insulation score varies depending on the identity of the bound insulators (Fig. 2F). For example, boundaries occupied by [BEAF-32, CTCF, & CP190] or by [CTCF, Su(Hw), & CP190] have weaker insulation than those bound by [BEAF-32, Su(Hw), & CP190] (Fig. 2F). Similarly, insulation strength varies in boundaries occupied by different combinations of two insulator proteins depending on their identity: For example, the median insulation of [BEAF-32 & CP190]–occupied boundaries is between the medians of the triple combinations [BEAF-32, CTCF, & CP190] and [BEAF-32, Su(Hw), & CP190]. CP190-only boundaries are an interesting exception, which have almost as strong insulation as those bound by all four proteins (Fig. 2F), suggesting that other CP190 recruiters are important for insulation at this stage of embryogenesis.

In summary, our results indicate a strong diversity in the occupancy of TAD boundaries by combinations of insulator proteins at NC14. The binding of more proteins has a higher likelihood that a site will form a boundary. However, it is not simply the number of bound insulator proteins that is the sole determinant of insulation; the nature of the bound factors (and presumably the genomic context) influences the strength of insulation at a boundary.

### Genetic depletion leads to very efficient removal of insulator proteins from chromatin in early embryos

To investigate the functional contribution of each insulator protein to the establishment of TADs and gene expression in early embryos, we removed the maternal deposition of each protein in females in the developing oocyte. For CTCF, we used our previously generated knockout allele that completely removes both the maternal and zygotic CTCF mRNA and protein (*38*). Genetic knockout of BEAF-32, Su(Hw), or CP190 leads to strongly reduced fertility, sterility, or homozygous lethality, respectively (*53*–*55*). We therefore used an alternative depletion strategy, through RNA interference (RNAi)–mediated knockdown in the female germ line (*56*). We could successfully obtain embryos after knockdown of maternal BEAF-32 or CP190 in the female germ line. Unfortunately, knockdown of maternal Su(Hw) led to female sterility, and we could therefore not obtain embryos to study the contribution of Su(Hw) in the establishment of TADs. We therefore focused on the role of CTCF (using genetic deletion of the maternal and zygotic contribution) and BEAF-32 and CP190 (by RNAi knockdown).

**Fig. 3.**
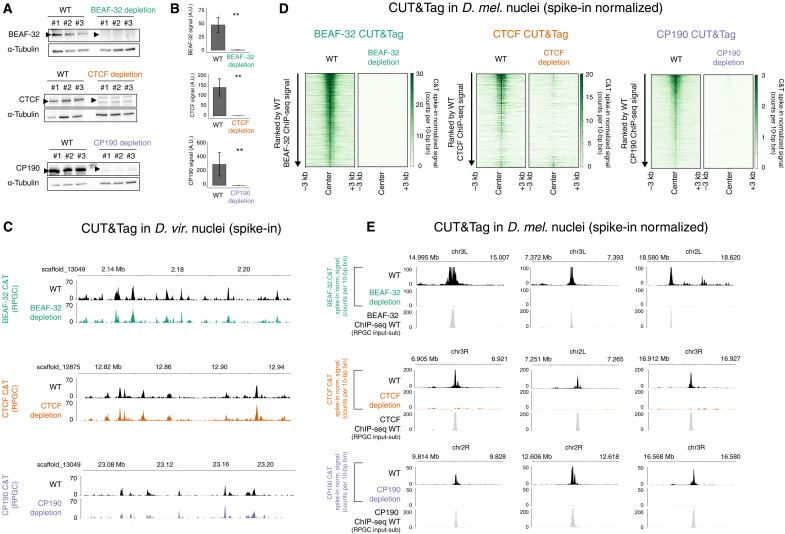
Efficient depletion of insulator proteins from chromatin. (**A**) Western blots for BEAF-32 (top), CTCF (middle), and CP190 (bottom) comparing three biological replicates of NC14 WT versus insulator-depleted embryos after maternal depletion of each insulator protein. BEAF-32, CTCF, and CP190 bands indicated by an arrow. α-Tubulin was used as a loading control to normalize all experiments. (**B**) Quantification of the Western blots, showing almost-complete depletion. Two-tailed *t* test, **P < 0.001. A.U., arbitrary units. (**C**) Genome browser showing *D. virilis* C&T signal (RPGC) at representative regions from experiments where nuclei were pooled (spiked-in) with *D. melanogaster* WT and insulator-depleted nuclei. In both samples, the *D. virilis* insulator peaks are present, confirming that the C&T worked. (**D**) Heatmaps of C&T normalized signal (with *D. virilis* spike-in rescaling) in *D. melanogaster* nuclei of WT and insulator-depleted samples from NC14 embryos, centered on the ChIP-seq peak summits for each protein. Heatmaps are ranked by C&T peak signal in WT embryos; (left) BEAF-32 C&T, (middle) CTCF C&T, and (right) CP190 C&T. (**E**) C&T signal (after *D. virilis* rescaling) in *D. melanogaster* showing representative regions in WT and insulator-depleted embryos. ChIP-seq in WT embryos shows peaks colocalized to C&T signal. These representative loci show the clear absence of insulator chromatin binding in depleted embryos compared to WT.

The efficiency of protein depletion in NC14 embryos was assessed by two metrics: first, by Western blot, which indicates that the three insulator proteins (BEAF-32, CTCF, or CP190) are globally very strongly depleted to almost undetectable levels (Fig. 3, A and B); second, by quantitative CUT&Tag (C&T) using spike-ins to determine the protein’s depletion from chromatin. For both WT and insulator-depleted embryos, C&T was performed on 50,000 *Drosophila melanogaster* nuclei (isolated from NC14 embryos) combined with 50,000 nuclei from another *Drosophila* species (*Drosophila virilis*) (isolated from 2- to 4-hour embryos). This *D. virilis* spike-in was added to every sample (both WT and insulator-depleted *D. melanogaster* nuclei) to control for the efficiency of tagmentation between samples and thereby enables a more accurate quantification of the reduction in ChIP peaks. Such spike-in controls are particularly important in cases like this where we expect almost a complete loss of binding in the depletion condition.

The occupancy profiles from the spiked-in *D. virilis* nuclei for all three insulator proteins were nearly identical for nuclei pooled with *D. melanogaster* WT and insulator depletion–matched samples, indicating that all C&T experiments worked efficiently (Fig. 3C). In contrast, in *D. melanogaster* nuclei, there was a marked reduction in chromatin binding for BEAF-32, CTCF, and CP190 in their respective depletion conditions (Fig. 3, D and E), while the binding was still present in the pooled *D. virilis* nuclei (Fig. 3C). We could detect almost no significant peaks overlapping any of the three insulator proteins’ WT peaks following their depletion (Fig. 3, D and E). This is important, as even low levels of insulator protein are enough to sustain chromatin topology in other models (*17*, *57*). We did observe some nonspecific peaks in the insulator-depleted samples not present in WT conditions. As these were only present after the depletion of the protein (i.e., in the *D. melanogaster* nuclei) and not in the *D. virilis* nuclei, this is likely to be spurious Tn5 activity (ATAC-seq-like signal) in the absence of the correct epitope for the primary antibodies. This is purely a technical artifact of C&T, while the binding of BEAF-32, CTCF, and CP190 at endogenous WT peaks as measured by ChIP-seq is severely reduced or completely absent (Fig. 3, D and E). These results confirm that our depletion conditions are very efficient at removing these factors from their bound regions and therefore can be used to assess the function of BEAF-32, CTCF, and CP190 in the establishment of TADs.

### Insulator depletion does not inhibit TAD formation but does weaken specific boundaries with different properties

**Fig. 4.**
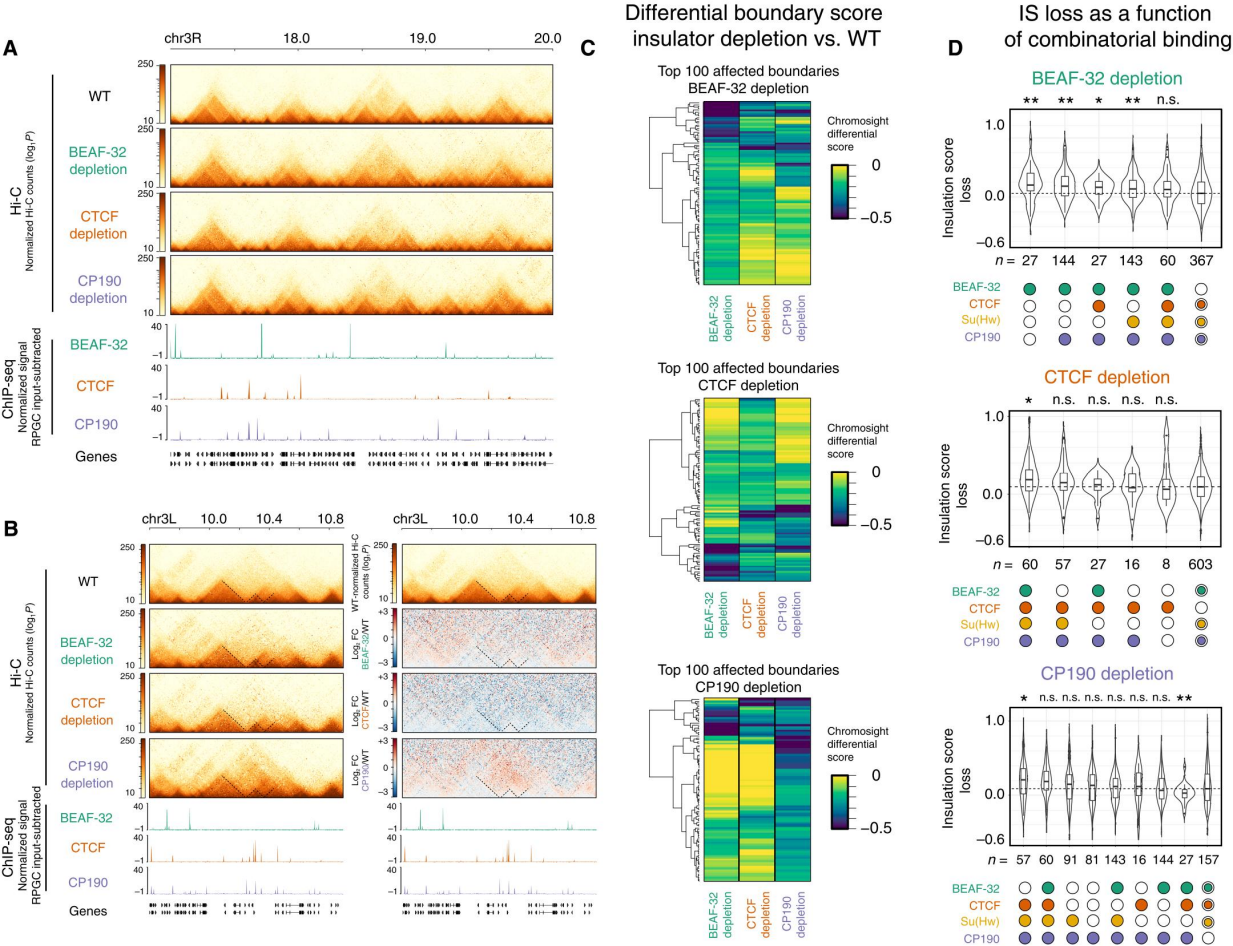
Depletion of insulator proteins has little global impact on the establishment of TADs during ZGA. (**A**) Hi-C matrices from WT and insulator-depleted embryos (2 to 3 hours), normalized by read counts, showing a representative region on chr3R. Underneath, ChIP-seq signal (RPGC input subtracted). (**B**) Left: Hi-C matrices of 1 of the top 100 regions with a weakened TAD boundary in CP190-depleted embryos. Right: Differential Hi-C signal [log_2_ fold change (FC)] of the same region, where the Hi-C signal (interaction frequencies) from the insulator-depleted samples is divided by the WT signal. Red, higher signal in depleted samples; blue, higher signal in WT embryos. (**C**) Heatmaps of the differential scores (Chromosight) (*58*) of observed changes at TAD boundaries in insulator-depleted versus WT embryos, for the top 100 weakened boundaries per genotype. Negative values (blue) indicate weakened boundary in comparison to WT, while zero (yellow) indicates no change. (**D**) Distribution of insulation score (IS) loss at TAD boundaries in BEAF-32–, CTCF-, and CP190-depleted embryos (top, middle, and bottom, respectively) compared to WT embryos at 2 to 3 hours. Plots ordered by the highest loss of insulation (left) to the smallest (right). Boundary occupancy by insulator proteins at NC14 (in WT embryos) shown underneath. Boundaries not occupied by the depleted insulator shown on the right. Kolmogorov-Smirnov, two-sided test. n.s., not significant. *P* > 0.05, *P < 0.05, and **P < 0.01.

We performed Hi-C in tightly staged 2- to 3-hour embryos (which are highly enriched for NC14) that were maternally depleted of each individual insulator protein, as described above. Unexpectedly, depletion of either BEAF-32, CTCF, or CP190 did not prevent the establishment of the majority of TADs, as observed in the Hi-C matrices (Fig. 4A). A similar number of TADs are detected across all genotypes (fig. S2B), which was robust to different TAD calling parameters. Chromosomal interactions across genomic distances are also similar for all genotypes for both WT and the different insulator-depleted embryos (fig. S2C). However, quantifying the insulation scores of TAD boundaries revealed a subtle genome-wide decrease in insulation (i.e., an increase in insulation score) in all three depletions (fig. S2D), although most boundaries are still detectable. Moreover, all three depletions are more highly correlated between each other compared to the WT replicates (fig. S2E). Similarly, quantifying interaction frequencies within TADs and across the boundary with the neighboring TADs (intra- versus inter-TAD interaction frequencies) revealed that all three insulator depletions have higher inter-TAD interactions compared to WT (fig. S2D), again suggesting weakened boundaries. However, although significant, this difference between the depletions and WT is subtle.

Despite these subtle genome-wide trends, some individual loci are more strongly affected. For example, some loci have weakened boundaries in specific genotypes, which are observed as gains of interactions across the TAD boundary, as seen in the differential Hi-C matrices (Fig. 4B). To systematically identify regions exhibiting differential interactions (disrupted TAD boundaries), we used Pareidolia, which extends the computer vision–based algorithm Chromosight (*58*), for differential analysis between two conditions. This identified 148 of 878 (17%), 119 of 869 (14%), and 151 of 878 (17%) boundaries with differential interactions (using a cutoff of <−0.1) between WT and BEAF-32–, CTCF-, or CP190-depleted embryos, respectively (table S1). This changed to 43 (4.9%), 43 (4.9%), and 81 (9%) boundaries using a more stringent cutoff of <−0.2 (table S1). Clustering the top 100 disrupted boundaries in each genotype shows the highest reduction in differential boundary score (from Chromosight) for that protein’s depletion, as expected (Fig. 4C, green/blue). This also revealed a number of boundaries whose insulation is affected by more than one genotype (Fig. 4C, green/blue). We complemented this with a visual curation of all affected TAD boundaries. A large fraction of the computationally identified top 100 affected TAD boundaries have an obvious alteration in the contact matrix by visual inspection (60, 52, and up to 75 for BEAF-32, CTCF, and CP190 depletions, respectively; table S1). This represents roughly 6 to 10% of all TADs. CP190 depletion had the strongest effects on boundary loss (as seen in the example in Fig. 4B and subsequent figures), in agreement with recent findings at later stages (*28*) and consistent with its presence at ~80% of all boundaries (Fig. 2) and its proposed insulator cofactor role.

To assess whether the TAD boundaries that are sensitive to different insulator protein depletion have specific features, we divided boundaries into different classes based on their combinatorial insulator binding (as in Fig. 2) and analyzed which classes had the largest loss in insulation score. The violin plots are ordered from the largest loss of insulation (greatest change in insulation score; Fig. 4D, left) to boundaries that are not bound by that insulator protein for comparison (Fig. 4D, right). This revealed that depletion of BEAF-32, CTCF, or CP190 led to a stronger reduction in insulation at domain boundaries occupied by these respective factors (note that a higher insulation score loss indicates a larger decrease in boundary insulation; Fig. 4D), which, although expected, confirms the specificity of the depletions. It also revealed interesting differences between BEAF-32 and CTCF and CP190. The boundaries that are only bound by BEAF-32 (or [BEAF-32 & CP190]) are the most severely affected after BEAF-32 depletion (Fig. 4D), suggesting that at these boundaries, BEAF-32 is essential for full insulation. However, at other boundaries co-occupied by BEAF-32 and other factors, BEAF-32 depletion has little impact (Fig. 4D), suggesting that, in these contexts, it acts redundantly. Conversely, depletion of CTCF or CP190 has stronger effects on combinatorially bound boundaries. For example, depletion of CTCF had a stronger reduction in insulation at boundaries bound by all four insulator proteins, while CP190 had a stronger impact on boundaries occupied by [CTCF, Su(Hw), & CP190], suggesting that both insulators function by more cooperative interactions (Fig. 4D). This was also evident when plotting the quantitative signal of insulator cobinding at affected boundaries upon BEAF-32, CTCF, or CP190 depletion (fig. S3A). This suggests that CTCF and CP190 act more cooperatively with other factors. For CTCF, at least part of this cooperativity is likely through CP190 itself, as CTCF was recently shown to be essential for CP190 recruitment at a subset of boundaries at later stages of embryogenesis (*29*). Within our top 100 boundaries affected by CTCF depletion, 39 overlap CP190 peaks at both NC14 and later stages (*29*), ~50% (19 of 39) of which are dependent on CTCF for CP190 recruitment (at least at later stages) (*29*).

Differences between BEAF-32, CTCF, and CP190 are also apparent when looking at the relationship between the changes in insulation score and the number of ChIP peaks for that factor. TAD boundaries that overlap more BEAF-32 peaks (within the 10-kb window) in WT embryos are more dependent on BEAF-32 (i.e., had a larger reduction in insulation after depletion) (fig. S3B). However, this trend is not significant for CTCF and CP190 (fig. S3B), and there is no correlation between the ChIP peak height and insulator score for all three proteins (fig. S3C).

Examining the distance of an affected boundary to the closest insulator ChIP peak also revealed differences between each insulator protein. Boundaries affected in CTCF and CP190 are closer to a CTCF peak and further away from a BEAF-32 or CP190 peak in comparison to a random background of unaffected boundaries (fig. S3D). In BEAF-32 depletion, affected boundaries are further away from CP190 peaks than random but not significantly closer to BEAF-32 peaks. Boundaries affected in all three depletions were further from CP190 peaks (fig. S3D), which might be due to the widespread presence of CP190 at 80% of boundaries (and thus present in many nonaffected boundaries). In most cases, the significant difference in the distance distributions concentrates at about 50 kb from the TAD boundaries and is therefore difficult to reconcile with a simple direct relationship between loss of binding at the boundary and loss of boundary insulation, but again, it hints at a functional connection between CTCF and CP190.

Together, these results indicate that the initial establishment of TADs during *Drosophila* embryogenesis is generally robust to the loss of a single insulator protein. However, TAD boundary insulation is globally decreased, and some specific TAD boundaries are more sensitive to the loss of a given insulator protein than others. Our observations indicate that CP190 and CTCF function more combinatorially compared to BEAF-32, although BEAF-32 and CP190 cobind much more extensively throughout the genome. This suggests that these insulators act together in a more complex (or specific) manner than simple redundancy between any of the insulators. Specific combinations of insulator proteins are needed for full insulation at different boundaries.

### Depletion of insulator proteins leads to transcriptional defects through different mechanisms

**Fig. 5.**
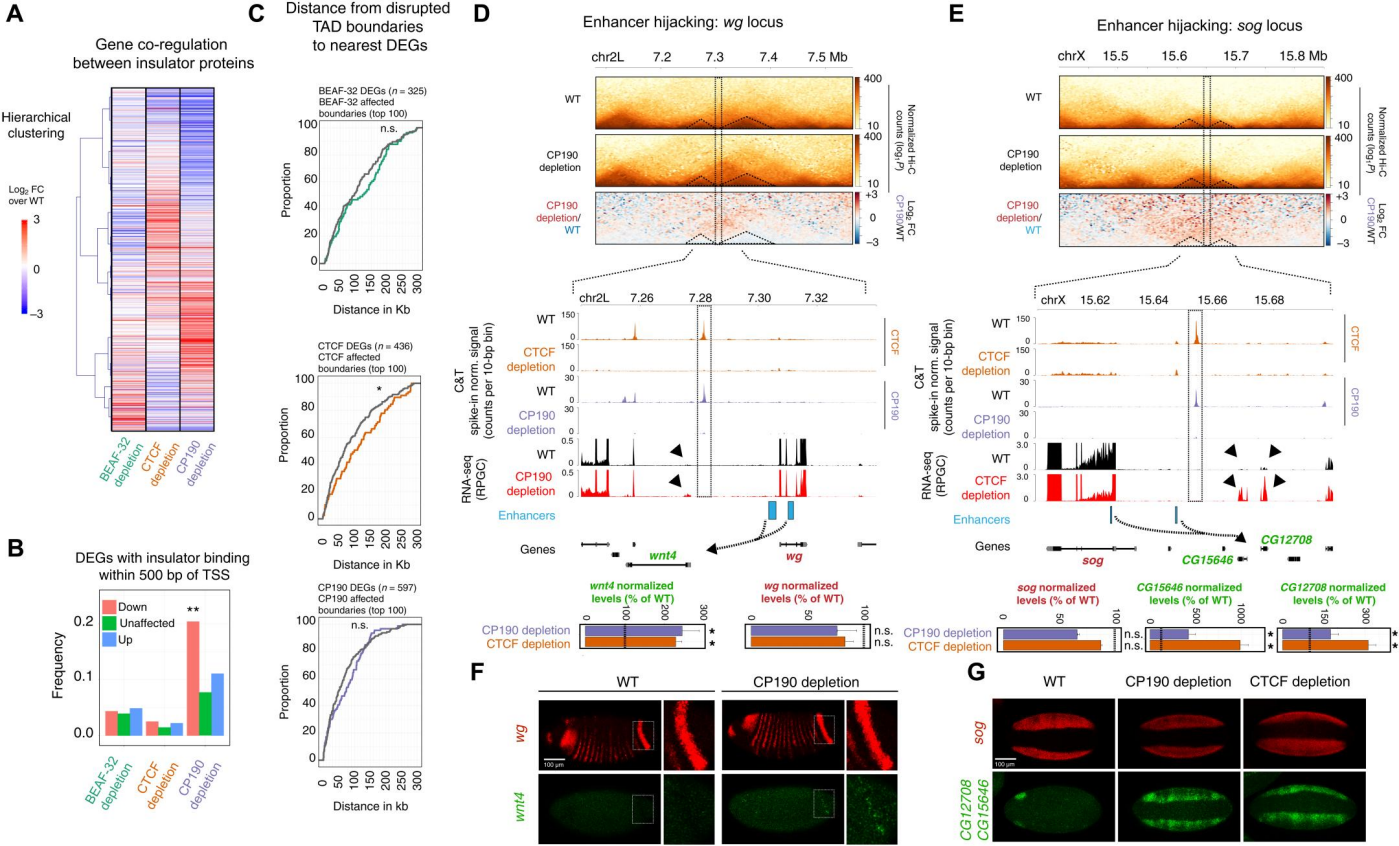
Transcriptional changes after insulator protein depletion. (**A**) Heatmap of RNA-seq (log_2_FC) in insulator-depleted versus WT NC14 embryos, with increased (red) and decreased (blue) changes (hierarchical clustering). Only zygotic DEGs in at least one genotype are included (1030 genes). (**B**) Proportion of DEGs in which the promoter (TSS ± 500 bp) overlaps a ChIP-seq peak for the corresponding insulator (in WT). Fisher’s exact test, **P < 0.0001. (**C**) Cumulative curves of the distance between DEG promoters to the nearest disrupted boundary (colored) or to nonaffected boundaries (gray). Kolmogorov-Smirnov, two-sided test, n.s. *P* > 0.05 and *P < 0.05. (**D** and **E**) Two loci displaying enhancer hijacking. Top: Hi-C and differential Hi-C matrices (log_2_FC) of the *wg* (D) and *sog* (E) loci. Bottom: Zoom-in of CP190 and CTCF occupancy (C&T) in WT and insulator-depleted NC14 embryos and RNA-seq signal (RPGC) from WT (black) and insulator-depleted (red) embryos (known enhancers, blue; TADs separated by a weakened boundary, dotted triangles; potential newly formed regulatory connections, dotted arrows). Genes highlighted in green are up-regulated in CP190 (*wnt4* and *CG15646*) or CTCF (*wnt4*, *CG15646*, and *CG12708*) depletion, while the enhancer’s original targets (red) are not affected. RNA-seq normalized levels are displayed in a bar plot; n.s. FDR > 0.05 and *FDR < 0.05. (**F**) In situ hybridization (ISH) of *wg* (red) and *wnt4* (green) in WT and CP190-depleted NC14 embryos. Inset highlights the posterior *wg* stripe, where a few cells in CP190-depleted embryos ectopically express *wnt4*. (**G**) ISH of *sog* (red) and *CG12708/CG15646* (green) in WT, CP190-depleted, and CTCF-deleted NC14 embryos. *CG12708/CG15646* genes are ectopically expressed in a similar pattern to *sog*. Scale bars, 100 μm.

To investigate how insulator protein depletion during the establishment of TADs affects gene expression, we performed RNA sequencing (RNA-seq) in manually selected embryos at NC14, in triplicates. Principal components (PC) analysis illustrates that in all three genotypes, PC1 clearly separates depletion and WT replicates (fig. S4A). To avoid any potential confounding trans-effects, we removed maternally deposited genes from the analysis [using RNA-seq data from unfertilized oocytes (*10*)] and thereby focused only on genes that started to be expressed at the ZGA. Examining these strictly zygotically expressed genes identified 325, 436, and 597 differentially expressed genes (DEGs) in BEAF-32–, CTCF-, and CP190-depleted embryos, respectively, compared to stage-matched WT embryos [|og_2_ fold change| > 0.7 and false discovery rate (FDR) < 0.05] (fig. S4B and table S2). This is largely balanced between the numbers of up- and down-regulated genes (fig. S4B). Around 25% of all DEGs (263 of 1030) have expression changes in the same direction in two of the three genotypes. Thus, the transcriptional responses following depletion of insulator proteins are largely genotype specific. However, CTCF and CP190 depletion resulted in slightly more overlap in misexpressed genes (Fig. 5A), in keeping with the similarity in their most affected TAD boundaries (discussed above; Fig. 4), and in the requirement of CTCF for CP190 recruitment at a subset of boundaries (*28*).

We also examined the expression of genes that are activated during the minor and major waves of ZGA (*59*) and are involved in the first spatial patterning events of the embryo. The majority of these genes had no significant changes in their mRNA levels after the depletion of BEAF-32, CTCF, or CP190, with a few exceptions including the up-regulation of the homeotic gene *scr* in CTCF mutant embryos (table S3). This indicates that ZGA and the activation of the majority of the anterior-posterior and dorsal-ventral patterning genes [some of which are regulated by known distant-acting elements, e.g., (*60*–*62*)] do not require these insulator proteins.

Insulator proteins have also been implicated in directly regulating gene expression (*63*, *64*). In *Drosophila*, for example, BEAF-32 (*30*) and CP190 (*31*) were proposed to have an activator role by directly binding to a subset of promoters, while CTCF was proposed to have either a transcriptional repressor (*65*) or activator (*17*, *64*, *66*) function. A subset of DEGs have insulator protein binding directly at their promoter [±500 base pairs (bp) from the transcription start site (TSS)] at this stage of embryogenesis (ZGA NC14 embryos) (Fig. 5B): 5% (15 of 325) of BEAF-32, 2% (10 of 436) of CTCF, and 16% (94 of 597) of CP190 DEGs. For BEAF and CTCF, these DEGs were roughly balanced between genes that were up- and down-regulated (7 of 15 and 4 of 10 down-regulated, respectively). However, 65% (61 of 94) of DEGs with CP190 binding at their promoter have reduced expression upon CP190 depletion (Fig. 5B). Therefore, our results show little evidence for direct promoter regulation by BEAF-32 or CTCF at these stages; however, CP190 may regulate the expression of a subset of genes more directly, independent of a role in TAD formation (Fig. 5B). This could involve the regulation of promoter-enhancer loops or a more direct regulatory role on the transcription machinery at the promoter. However, this represents only a small fraction (16% or less) of all misexpressed genes after CP190 depletion.

To explore the relationship between TAD boundary disruption and gene misexpression, we focused on the top 100 most affected TAD boundaries in each genotype and measured their distance to the nearest DEG (Fig. 5C). In all three depletions, the nearest DEG is located up to 300 kb away from a disrupted boundary, with a median distance of ~125, 100, and 70 kb for BEAF-32, CTCF, and CP190, respectively (Fig. 5C). This distance distribution is comparable to the background (i.e., the distance of 100 unaffected boundaries to the nearest DEG) [Fig. 5C, compare colored line (disrupted TADs) to gray (background)] and is larger than the average TAD size that most of these genes reside in. This indicates that DEGs are generally not enriched near disrupted TAD boundaries and that the TAD boundary is not constraining enhancer activity in the majority of cases, as we and others observed previously (*8*, *10*).

However, there are examples of DEGs close to the disrupted TAD boundary and therefore could be good candidates for enhancer hijacking, where an enhancer in one TAD leads to the misregulation of a gene in a neighboring TAD after disruption of the boundary that normally segregates the two. Such examples have been previously observed in both mammals (*9*, *11*, *67*) and *Drosophila* (*10*, *68*). Here, using this more rapid depletion, in a single generation, we searched for DEGs (zygotic only) with up-regulated expression in the neighboring TAD of TADs with affected boundaries after each depletion. Taking the top 100 affected boundaries in each genotype, we identified 10 (6%; 10 of 164 DEGs), 34 (12%; 34 of 275), and 40 (13%; 40 of 298) potential cases in BEAF-32, CTCF, and CP190 depletions, where a zygotic gene is up-regulated in the neighboring TAD (table S4). We chose three of these potential hijacking candidates that also have characterized enhancers that are active at NC14 to examine in more detail by fluorescent *in-situ* hybridisation (FISH). While one had no detectable change in expression (*hairy* locus), the other two (*wingless* and *short gastrulation*) had (Fig. 5, D and E).

*Wingless* (*wg*) is the ligand of one of the most characterized developmental signaling pathways whose expression initiates at NC14 to control the anterior-posterior patterning of the early embryo. *wg* and its enhancers are contained between two CTCF/CP190 binding sites, which separate them from the neighboring *wnt4* gene, which is only activated later in development (Fig. 5D). One of the CTCF/CP190 ChIP peaks overlaps a TAD boundary between *wg* and *Wnt4*. This peak is lost and the TAD boundary is disrupted after CP190 (and CTCF) depletion (Fig. 5D). Our RNA-seq experiments indicate no change in *wg* expression but a slight yet significant increase in *wnt4* expression, located in the neighboring domain upon CP190 depletion (Fig. 5D, arrowhead). Using RNA FISH, we observed a few cells that acquire *wnt4* misexpression, which largely colocalizes with the high levels of *wg* in a posterior stripe (Fig. 5F, inset) or in a patch in the anterior of the embryo. This ectopic expression pattern suggests that the *wg* enhancer(s) controlling this expression pattern start(s) activating the *wnt4* promoter upon loss of CP190.

An even more marked example is at the *short gastrulation* (*sog*) locus. *sog* is an essential gene expressed in two bands along the anterior-posterior axis of the embryo that will give rise to the neuroectoderm while being excluded from the presumptive mesoderm in between. Similar to *wg*, the *sog* locus is flanked by CTCF- and CP190-bound regions on both sides—the 3′ CTCF-CP190–bound region overlaps a disrupted domain boundary in both CTCF- and CP190-depleted embryos (Fig. 5E). Our RNA-seq measurements showed no alteration in *sog* expression but an up-regulation of two genes (*CG12708* and *CG15646*) in the neighboring domain on the right-hand side of the boundary, in both CTCF- and CP190-depleted embryos (Fig. 5E, arrowheads). In situ hybridization using a probe that overlaps both genes shows that they are normally only expressed in a small domain in the anterior end of the embryo in NC14 stage embryos (Fig. 5G). However, they become misexpressed in a *sog*-like pattern in both CP190- and CTCF-depleted embryos (Fig. 5G), again suggesting that a *sog* enhancer can now communicate with these genes’ promoters.

Together, our results indicate that these insulator proteins likely regulate gene expression through multiple mechanisms at these stages of embryogenesis. This includes potentially acting directly at the promoter to activate gene expression (2 to 20% of down-regulated genes) and by constraining enhancer activity at domain boundaries (6 to 13% of up-regulated genes), exemplified by enhancer hijacking at the *wg* and *sog* loci. In these hijacking cases, neither the promoters [of both the patterning genes and misexpressed genes (*wnt4*, *CG12708*, and *CG15646*)] nor the enhancers (or their surrounding regions) are bound by insulators, indicating that these insulators do not mediate enhancer-promoter tethering but rather modulate their communication indirectly, by maintaining the nearby boundary. However, both of these mechanisms can only account for a minority (<20%) of all DEGs. The majority of DEGs have no obvious direct relationship to changes in topology and likely represent indirect effects. DEGs are enriched in various metabolic and developmental processes, including transcriptional regulators (by gene ontology term enrichments), and secondary targets of these factors are likely among the remaining >80% DEGs. There are very few cases of DEGs that directly overlap (within 10 kb) a disrupted TAD boundary after insulator protein depletion. This indicates that TAD boundaries can be diminished while maintaining normal transcription of the housekeeping genes present at that boundary and also perhaps that redundancy between insulator proteins and transcription itself helps to maintain insulation at boundaries.

### An active promoter and an insulator-bound region are both required for full insulation of a TAD boundary

**Fig. 6.**
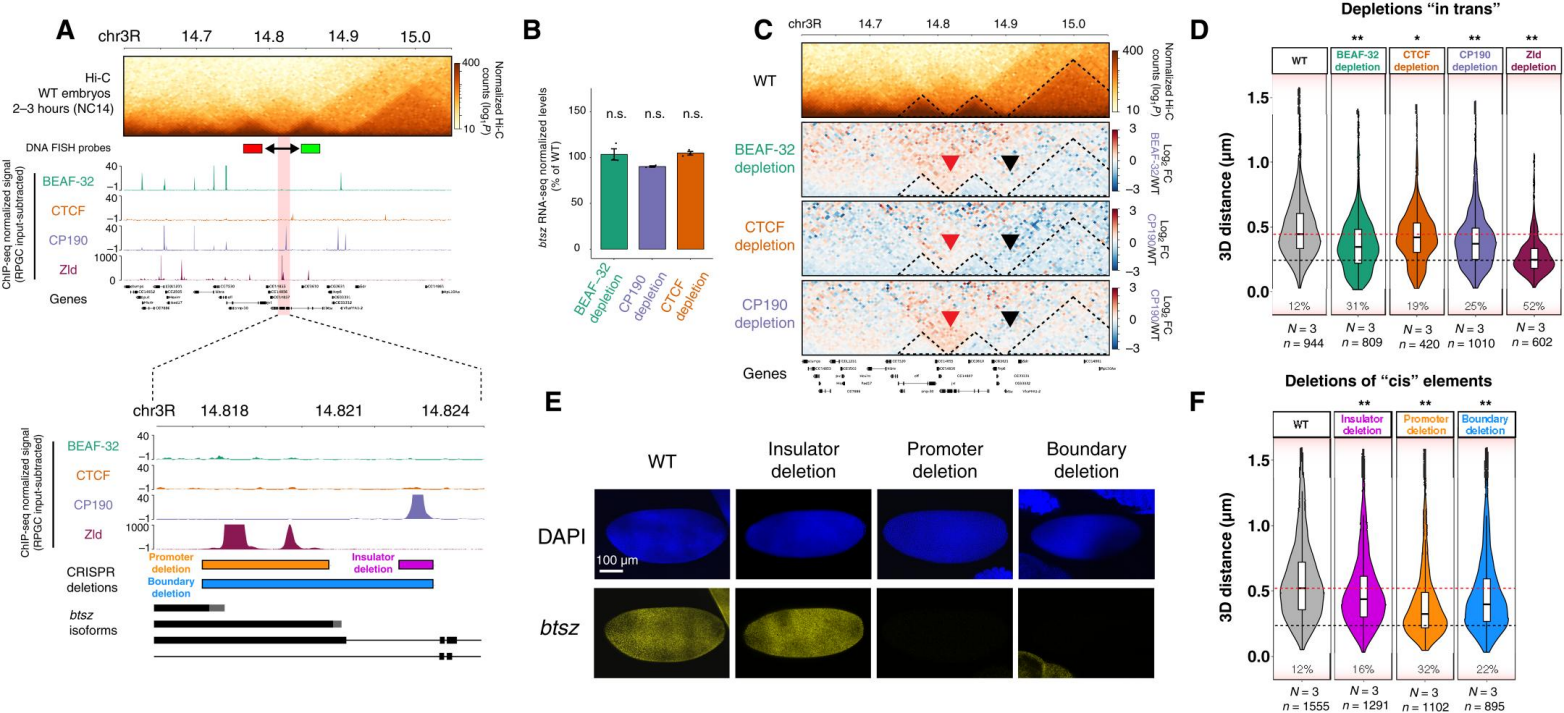
Deletion of an active promoter or a CP190 binding site affects insulation at the *btsz* TAD boundary. (**A**) Genomic features at the *bitesize* (*btsz*) locus. Top: Hi-C matrix from WT embryos (2 to 3 hours) and BEAF-32, CTCF, and CP190 occupancy (ChIP-seq from NC14 embryos) and Zld occupancy [from (*81*)]. Location of DNA-FISH probes indicated by red and green rectangles. Bottom: Zoom-in to occupancy at the TAD boundary and *btsz* promoter. Location of deleted regions indicated by colored rectangles. (**B**) Quantification of *btsz* RNA-seq signal in each insulator depletion (normalized to WT); n.s. FDR > 0.05. (**C**) Hi-C matrix from 2- to 3-hour WT embryos and differential Hi-C matrices (log_2_ fold change) from 2- to 3-hour embryos (higher in insulator protein depletion, red; higher in WT, blue). Red arrowhead indicates the disrupted *btsz* boundary, and black arrowhead indicates the neighboring unaffected boundary. (**D**) Quantification of 3D distances between the centers of mass of the DNA-FISH probes indicated in (A) across multiple alleles from WT and insulator-depleted embryos at NC14 (*N*, number of embryos; *n*, number of alleles). Percentages indicate the number of alleles with distances between the two probes below 250 nm (black dotted line). Median distances in WT embryos (red line). Kolmogorov-Smirnov test, *P < 0.01 and **P < 0.001. (**E**) ISH of *btsz* (yellow) in WT and CRISPR deletion embryos at NC14. DAPI in blue. Scale bar, 100 μm. (**F**) Quantification of 3D distances between the centers of mass of the DNA-FISH probes indicated in (A) in WT and CRISPR deletion embryos. *N*, number of embryos; *n*, number of alleles measured. Percentages indicate the number of alleles with probe distances below 250 nm. Dotted lines indicate distances at the WT median (red) and 250 nm (black). Kolmogorov-Smirnov test, *P < 0.01 and **P < 0.001.

To explore whether an active promoter at a boundary (i.e., either the active promoter itself or transcription) can add to the overall robustness of a boundary and thereby help maintain boundary insulation after depletion of insulator proteins, we functionally dissected one boundary. We selected a boundary that overlaps both an insulator-bound region and an active promoter that drives strong expression of the main isoform of the gene *bitesize* (*btsz*) during NC14 (*69*). This promoter is bound by the transcription factor Zld at NC14 (Fig. 6A), which is essential for *btsz* expression at this stage of embryogenesis (*21*, *70*). The boundary is also occupied by CP190 at NC14, which binds ~5 kb upstream of the *btsz* promoter (Fig. 6A). Both BEAF-32 and CTCF do not bind at the boundary, but CTCF binds to a site ~12 kb upstream of the promoter, while BEAF-32 cobinds with CP190 to the boundaries of the two adjacent domains (Fig. 6A). In contrast to Zld, there is no significant change in *btsz* expression upon depletion of any of the three insulator proteins (Fig. 6B).

To measure the impact of insulator protein depletion on boundary function, we used both Hi-C and DNA-FISH. Differential Hi-C maps indicate an increase in interactions crossing the domain boundary upon depletion of any of the three insulator proteins (Fig. 6C). This was confirmed independently using Chromosight (diff_score in relation to WT: −0.61, −0.48, and −0.49 for BEAF-32, CTCF, and CP190 depletions, respectively) and by calculating changes in insulation score (−0.71, −0.07, −0.04, and 0.13 for WT, BEAF-32, CTCF, and CP190 depletions, respectively). Concordantly, DNA-FISH measurements of the distance between the centers of the two adjacent domains (probes indicated in Fig. 6A) showed a decrease in domain distances (i.e., higher proximity) following insulator protein depletion: 12% of cells had these domains within 250 nm in WT embryos (Fig. 6D, bottom dashed line), which shifted to 31, 19, and 25% after the depletion of BEAF-32, CTCF, and CP190, respectively. Thus, both approaches indicate that removing any of the three insulators affects *btsz* domain boundary function, even if these insulators (BEAF-32 and CTCF) are not directly binding at the central domain boundary.

To assess the role of the active promoter, we first removed the major transcriptional regulator of this promoter, Zld (*21*, *70*). Zld depletion led to an even stronger loss of boundary function (52% of cells with distances of <250 nm; Fig. 6D), indicating that at this locus, the activation of the promoter is more important for boundary function than insulator binding. To confirm that these effects are due to regulation in *cis* and not to potentially secondary *trans*-effects, we used CRISPR-Cas9 to delete three elements within the region: (i) the *btsz* promoter and promoter-proximal region (~3.5 kb), (ii) the CP190-bound region (~1 kb) leaving the *btsz* promoter intact, and (iii) both the *btsz* promoter and CP190-bound region (a 6-kb deletion spanning the entire boundary) (Fig. 6A, bottom). Deletion of the promoter (~3.5 kb) or the entire boundary (~6 kb) completely abolished *btsz* expression in NC14 embryos, as expected, while the insulator deletion had no detectable effect on *btsz* expression, confirming that the promoter is still active (Fig. 6E). Accordingly, since *btsz* is an essential gene, the promoter and the entire boundary deletions are homozygous lethal, while flies with the insulator binding site deletion are homozygous viable and fertile. To measure the insulation across the domain boundary in these lines, we performed DNA-FISH on embryos at NC14 (similar to Fig. 6D). Deletion of the insulator binding site led to a reduction in insulation between the two domains as seen in their increased proximity: The number of cells with distances of <250 nm changed from 12 to 16% in the WT and insulator deletion, respectively (Fig. 6F, bottom dashed line). However, the changes were much more marked in embryos with the active promoter deletion (Fig. 6F), changing from 12% (WT) to 32% (promoter deletion) of cells with distances of <250 nm. Therefore, at this locus, deletion of the active promoter had a more pronounced effect on boundary function compared to deletion of the insulator-bound region itself (Fig. 6F).

In summary, although deletion of the insulator binding site in *cis* (Fig. 6F) and depletion of insulator proteins in *trans* (Fig. 6D) have an effect on boundary function, the deletion of the promoter region and its activator protein, Zld, also had a marked effect on boundary insulation. This suggests that an active promoter (or transcription or some other property of the occupied promoter) is required for full boundary function and acts together with insulator protein binding, perhaps to reinforce boundary insulation. Of the insulators tested, the depletion of BEAF-32 had the most prominent effect (Fig. 6D), although it is not bound to the central boundary. This indicates that insulator proteins can influence domain boundary function in a long-range manner at some loci, perhaps by perturbing the compaction of the neighboring domains.

## DISCUSSION

### Genomic regions with different insulator binding signatures form domain boundaries in *Drosophila*

In mammals, deletion of individual CTCF sites typically has limited effects on TAD insulation. The fusion between two TADs usually requires deletion of multiple CTCF sites (*12*, *52*, *71*). In *Drosophila*, we observed, in line with others, that TAD boundaries are typically occupied combinatorically by multiple insulator proteins, which can be a mixture between multiple peaks for the same factor (homotypic) and multiple peaks of different factors (heterotypic) binding: e.g., for NC14, see Fig. 2, and for later embryonic stages, see (*4*, *23*–*26*). For example, we observe both insulators cobinding at a single site (i.e., within a 200-bp window; Fig. 2C, left), consistent with the recruitment of CP190 by either BEAF-32 or CTCF (*29*, *31*, *43*, *48*), in addition to more distributed binding within the same boundary region (Fig. 2C, right), with up to four different peaks for the same factor within a 10-kb boundary region. This suggests a potential basis for the robustness of TAD boundaries—perhaps the loss of a single factor is not enough to completely abolish boundary function. However, our data indicate that the strength of insulation is not simply dependent on the number of different cobound insulator proteins. In other words, this is not simple redundancy but rather the identity of the combination of factors present at each boundary matters. Different combinations of factors are associated with boundaries of different insulation strengths, with BEAF-32 appearing to act more redundantly, while CTCF and CP190 may act more cooperatively between each other and with other factors (Fig. 4D). These results highlight the diversity of *Drosophila* boundaries and the difficulty to predict whether an insulator-bound site will be able to form a boundary or not.

### Insulator proteins are individually not required for the formation of most TADs

Our depletion strategy allowed us to achieve near-full depletion of these insulator proteins from chromatin, so they are not present at the moment when TADs are being established (Fig. 3). Using C&T with spike-ins was crucial to confidently and quantitatively compare insulator protein binding between control and insulator-depleted embryos. This revealed that there are no statistically significant peaks identified, of the thousands of WT peaks, in insulator-depleted embryos. This is important, as even low levels of insulator protein are enough to sustain chromatin topology in other models (*17*, *57*).

In the absence of the insulator proteins BEAF-32, CTCF, or CP190, we observed that most *Drosophila* TADs (~90%) can still form with little apparent changes (Fig. 4), although 86% of TAD boundaries are occupied by at least one of these three proteins (Fig. 2). This robustness may be explained by alternative, not necessarily exclusive, hypotheses. One commonly proposed mechanism is redundancy between these insulator proteins, as multiple different proteins have been identified in *Drosophila*, as reviewed in (*22*). In mammals, TADs are formed by cohesin-mediated loop extrusion, which stalls at CTCF-bound sites (*15*, *16*). Although there is currently no experimental evidence for cohesin-mediated loop extrusion in *Drosophila*, cohesin (or another loop extruder) could, in principle, be stalled by any of the C2H2 zinc finger insulator proteins in *Drosophila*, including CTCF. As we removed each one of these three insulator proteins individually, another factor could have compensated for their loss and facilitated stalling of loop extrusion to create the TAD boundary. Our data suggest that at each boundary, this is not simply the presence of “any” other insulator—there appears to be some specificity, as different boundaries have different sensitivities to each insulator protein’s depletion (Fig. 4D).

Alternatively, as the majority [almost 80% according to Ramirez *et al.* (*23*)] of *Drosophila* TAD boundaries overlap an active promoter of constitutively expressed genes (*4*, *23*, *34*), an active promoter (or transcription) may be involved in maintaining TAD boundary function. The injection of pharmacological inhibitors that block transcription had little apparent impact on the establishment of TADs at this stage (*21*, *72*). However, our data dissecting the function of one boundary at the *btsz* TAD indicate that an active promoter (or transcription) is required for full boundary insulation. Deleting the promoter region had a stronger impact compared to deleting the insulator-bound region itself (Fig. 6, D and F). This is suggestive of a role of active promoters (or transcription) in boundary insulation, although we cannot exclude that the functional “boundary entity” is not polymerase II occupancy at the promoter (or formation of a preinitiation complex) or Zld binding itself, as discussed below. This promoter-associated function is also not the only requirement for boundary function (i.e., it does not abrogate insulation completely), but perhaps, it plays a role in the reinforcement of TAD boundaries after they are formed.

Last, TAD boundaries may be strongly locus specific in *Drosophila*, i.e., each boundary may rely on a specific combination of factors in addition to the surrounding genomic context to function. This is supported by the different subsets of boundaries that are more sensitive to depletion of different factors, as we observed here during ZGA and as has also been observed in other studies at different stages/contexts (*23*, *28*, *29*, *38*, *39*, *73*). For example, for the factors depleted here, most disrupted boundaries were genotype specific and affected in the range of 6 to 10% of all TADs. Nevertheless, boundaries affected by depletion of CTCF and CP190 had more overlap compared to BEAF-32 depletion (Fig. 4). Even for the most affected TAD boundaries, the absence of the insulator protein did not lead to a complete loss of the boundary or complete fusion between neighboring TADs but rather a weakening of the boundary. As TADs are just forming at this stage and still quite fuzzy (due to extensive cell-to-cell variation; Fig. 1), we assumed that they would be easier to disrupt or break, but this does not seem to be the case. They are seemingly as resilient to perturbation here as seen in other contexts (*12*, *23*, *28*, *29*, *38*, *39*, *52*, *73*).

### Insulator proteins contribute to gene expression through different mechanisms

By definition, the major event that is occurring during ZGA is the activation of transcription in the embryo, making this a very interesting stage to examine the requirement of these proteins for both chromatin topology and the initiation of gene expression. After the depletion of each of the three factors, a few hundred zygotically expressed genes had significant changes in their expression (Fig. 5). However, it is interesting to note that this did not include many of the “classic” minor and major wave early patterning genes (*59*). However, there are some exceptions: (i) 1 of 10 pair-rule genes [*even-skipped* (*eve*)] was slightly down-regulated in CP190 depletion, and (ii) 2 of 8 homeotic genes had a change in expression: *Scr* was slightly up-regulated in BEAF-32 and down-regulated in CTCF depletions, while *Ultrabithorax* (*Ubx*) was down-regulated in CP190 depletion (table S3). None of the 13 gap genes were misregulated in any genotype. Therefore, ZGA can still occur largely unperturbed after the depletion of each of these insulator proteins, despite their occupancy at the majority of TAD boundaries and thousands of intra-TAD sites.

These insulator proteins may regulate a small fraction of genes (from 2 to 20% of the down-regulated genes in each genotype) by directly binding at their promoter, which can be due to the regulation of transcriptional initiation or by mediating communication with other cis-regulatory elements. We also identified a small number of cases of enhancer hijacking (6 to 13% depending on the genotype), where an enhancer in one TAD with a weakened boundary misappropriately activates a gene in the neighboring TAD (Fig. 5). For example, in the *wg* and *sog* loci, the TAD boundary seems to have restricted the enhancer’s activity such that weakening the boundary leads to enhancer activation of an additional target gene in the neighboring TAD. It is interesting to note that such enhancer hijacking cases varied in their effect size. In the *wg* locus, for example, disruption of the boundary is associated with a gain of *wnt4* expression in a very small number of cells, while boundary disruption at the *hairy* locus had no obvious effect on the neighboring gene’s expression. In comparison, disruption of a boundary at the *sog* locus led to *CG12708*/*CG15646* misexpression in the majority of *sog*-expressing cells. These observations, together with recent studies, indicate that the impact of boundary perturbations on gene expression, if any, depends on multiple factors (*74*). However, together, both mechanisms (direct promoter regulation and enhancer hijacking) can only account for a minority (<20% at best) of all misexpressed genes after these insulators’ depletion. DEGs are generally not enriched near disrupted TAD boundaries; the median distance of misexpressed genes is ~125, 100, and 70 kb from a disrupted boundary for BEAF-32, CTCF, and CP190 depletions, respectively (Fig. 5C). This suggests that these genes change in expression by secondary effects, perhaps by misregulated transcription factors or by other, as yet not understood mechanisms.

### A potential role of active promoters or transcription in boundary strength

Using genetic deletions, we dissected one complex TAD boundary that simultaneously overlaps an active promoter and an insulator-bound region during ZGA (Fig. 6). In that boundary, deleting the active promoter (or removing its direct activator, Zld) caused a stronger effect on boundary insulation, in comparison to deleting the insulator binding site, which still had an effect on boundary insulation. This indicates that the boundary requires both insulators and the active promoter region for full insulation strength. At this time, it is still unclear what endows the active promoter region with “boundary activity.” It could be the assembly of a preinitiation complex, which is of substantial size (estimated ~100 proteins), and may therefore be sufficient to confer insulation by, for example, blocking loop extrusion. Alternatively, the formation of a transcription bubble, which is associated with local negative supercoiling, could also provide insulation, perhaps by blocking or slowing down loop extrusion. Paused polymerase could also play a role, as paused promoters can have insulator-like activity in enhancer-blocking transgenic-reporter assays (*75*), or perhaps, it is the movement of polymerase during transcriptional elongation. Many insulator proteins, including CTCF in mammals, have been proposed to also act as “normal” transcriptional activators. Although very speculative, this activation of transcription at promoters could also serve to reinforce these proteins’ ability to insulate regulatory domains.

Together, our results suggest that *Drosophila* domain boundaries are established in part by different combinations of insulator proteins, which are influencing the topology of each locus to a different degree. Approximately 80% of *Drosophila* boundaries contain promoters, and an active promoter or transcription at the boundary may also influence boundary strength as we show here for the *btsz* boundary. Future studies are needed to understand the relationship between insulator combinatorial binding and their genomic context to dissect the rules that govern the formation of domain boundaries in *Drosophila* and the cross-talk between boundary function and active promoters (or transcription).

## MATERIALS AND METHODS

The Western blots, FISH, Hi-C, RNA-seq, ChIP-seq data processing, RNA-seq analysis, and Hi-C data analysis were performed with standard procedures. Detailed methods for each are provided in the Supplementary Materials, while the more specific methods used in this study are provided below.

### Genetic deletion/knockdown of CTCF, BEAF-32, and CP190

Maternal knockout of CTCF depletion was performed as described previously in (*38*). Briefly, CTCF knockout flies were rescued with an FRT-flanked 5-kb CTCF genomic rescue transgene and developed into viable and fertile adults. The excision of the CTCF rescue cassette from male and female germ lines was achieved through nanos-GAL4:VP16 (NGVP16)–driven expression of UAS-FLP, and the resulting maternal/zygotic CTCF-depleted embryos were collected. This thereby generates complete genetic loss-of-function embryos.

BEAF-32 and CP190 depletion was performed by RNAi-mediated knockdown using stocks carrying short hairpin RNAs (shRNAs) as described previously (*56*). We first compared the efficiency of different germline GAL4 drivers and different temperatures. Virgin females carrying either BEAF-32 or CP190 shRNA [Vienna Drosophila Resource Center (VDRC), #330274 and Bloomington Drosophila Stock Center (BDSC), #33903, respectively] were crossed to males from either the MTD-Gal4 (BDSC, #31777) or Mat-tub-Gal4 lines (BDSC, #7063). These GAL4 lines express GAL4 either during all stages of oogenesis (MTD-Gal4) or only during late stages (Mat-tub-Gal4) (*56*). The crosses were incubated at 25° or 29°C, leading to different GAL4 efficiencies. F_1_ virgin females carrying one copy of either the BEAF-32 or CP190 shRNAs and one copy of the GAL4 transgene(s) were crossed to males carrying the BEAF-32 or CP190 shRNA, respectively. F_2_ embryos were collected and used for all experiments. For BEAF-32 depletion, the optimal combination was the mat-tub-GAL4 driver and 25°C, as other conditions (either the MTD-gal4 driver or the 29°C temperature) highly increased sterility. For CP190 depletions, the MTD-Gal4 driver and 29°C were used as this resulted in stronger levels of protein depletion (assessed by Western blot).

### CRISPR deletions in the *btsz* locus

To generate flies with CRISPR deletions (Fig. 6), CRISPR donor and guide RNA (gRNA) plasmids were constructed following the strategy for “gene replacement with pHD-DsRed-attP” described in (*76*). gRNA sequences were generated by annealed oligo cloning and inserted into the Bbs I site of the pU6-BbsI-gRNA vector. To generate the homology arms, we polymerase chain reaction (PCR)–amplified regions between 2 and 3 kb starting directly from the upstream or downstream cutting site of each gRNA and inserted those into the Sap I and Aar I sites of the pHD-DsRed-attP vector, using the In-Fusion cloning kit (#639650, Takara Bio USA Inc.). Primers and gRNA oligos are listed in table S5. Both gRNA and homology repair template plasmids were injected into embryos carrying a *vasa-Cas9* transgene on the third chromosome (BDSC #51324, w[1118]; PBac{y[+mDint2]=vas-Cas9}). Hatching adults were crossed to flies carrying a third chromosome balancer marked by green fluorescent protein (GFP), and the progeny was screened for both the DsRed and GFP markers. PCR genotyping was used to confirm the editing. DsRed^+^/GFP^+^ siblings were crossed, and the progeny was assessed for viability. Flies carrying a disruption of the *btsz* promoter or the whole boundary were not able to be maintained as homozygous stocks and were therefore maintained as a transheterozygous stock over a marker balancer chromosome.

### Embryo collections

Freshly hatched adults were placed in embryo collection vials with standard apple cap plates. For DNA-FISH experiments, following three 1-hour pre-lays, the flies were allowed to lay for 3 hours, and embryos were directly collected. For genomic experiments, following three 1-hour pre-lays, the flies were either allowed to lay for 30 min after which the embryos were aged for 2h10 to reach the interval 2 hours and 10 min. - 2 hours and 40 min. (ChIP-seq at NC14), or the flies were allowed to lay for 1 hour, after which the embryos were aged for 2 hours to reach the interval of 2 to 3 hours (C&T, Hi-C, Western blot, and RNA-seq). The embryos were then dechorionated using 50% bleach and washed with deionized water and PBT 0.1% (phosphate-buffered saline containing 0.1% Triton X-100). Embryos used for FISH were cross-linked in 4% formaldehyde for 20 min at room temperature, devitelinized, and stored in 100% methanol at −20°C. Embryos used for Western blot and RNA-seq were kept on ice-cold PBT, and NC14 embryos were manually selected using an embryo needle, based on morphological indicators (*77*) under a stereoscope, and then directly placed in sample buffer (Western blot) or snap-frozen in liquid nitrogen (RNA-seq). Embryos used for ChIP-seq, C&T, and Hi-C (2 to 3 hours) were cross-linked in 1.8% formaldehyde for 15 min or 3% formaldehyde for 30 min at room temperature. Fixation was stopped by addition of PBT 0.1% + 125 mM glycine, followed by a wash with PBT 0.1%, then air-dried on tissue, and snap-frozen in liquid nitrogen.

### 3D DNA-FISH and combined DNA/RNA FISH

DNA-FISH probes were generated by PCR amplification from *D. melanogaster* genomic DNA (7- to 8.5-kb fragments) and TA cloning into a PGEMT-Easy vector (Promega, #A1360) (primers listed in table S5), except for the *btsz* locus, for which bacterial artificial chromosomes were used from DGRC (CHORI22-94I16 and CHORI22-115C08). The Nick Translation kit (Abbott Bioscience, #7J0001) was used to fluorescently label probes.

Embryos fixed with 1.8% formaldehyde and stored in 100% methanol were rehydrated, washed three times in 2× SSCT (2× SSC + 0.1% Tween), and then washed once in 20% formamide and in 50% formamide (both in 2× SSCT) at room temperature on a rotating shaker. This was followed by two 1-hour washes in 50% formamide at 37°C while rotating. The 50% formamide was removed, and the embryos were denatured at 80°C for 15 min in a water bath, placed on ice, and mixed with hybridization mix containing the fluorescent DNA probes. Following overnight hybridization at 37°C, embryos were washed twice in 50% formamide while rotating at 37°C, 1× with 20% formamide, and 3× in SSCT while rotating at room temperature. For DNA-FISH using a fluorescent marker (*btsz* CRISPR deletions; Fig. 6), the protocol continued using the HCR RNA FISH (Molecular Instruments) protocol to detect the GFP balancer chromosome, following the manufacturer’s instructions. Embryos were placed in ProLong Gold mounting medium with 4′,6-diamidino-2-phenylindole (DAPI; Thermo Fisher Scientific, #P36931) and mounted onto a slide.

Slides were imaged using a Leica SP8 confocal microscope with 100× objective (HC PL APO CS2 100×/numerical aperture 1.4/oil), a 405-nm laser, a white-light laser (470 to 670 nm), and HyD detectors. The z-stack step size was 200 nm. For all DNA-FISH samples, we acquired z-stacks covering a single layer of nuclei in the center of NC14 embryos. At least three embryos and hundreds of alleles were used per condition.

To precisely stage each single embryo, the number of nuclei in a 50-μm^2^ window was counted in each image, according to (*78*). Images were deconvolved using the Huygens Professional software (SVI) with default parameters. For the quantification of distances between DNA-FISH probes in each image, we used a custom FIJI plugin (“analyze FISH spots”) developed in-house by the European Molecular Biology Laboratory (EMBL) Advanced Light Microscopy Facility. The plugin has two main functions: (i) detect spots across different channels and (ii) automatically calculate the three-dimensional (3D) distances between spots. Briefly, the *x*, *y*, and *z* coordinates of FISH spots are determined on the basis of a manually provided value for signal intensity and background in each channel following visual inspection. The plugin displays the spots in the image, and the multipoint tool is used to manually select “clusters” of nuclear spots within a nucleus in the different channels along the z-stack (two or three channels depending on the number of DNA-FISH probes used). After all spot clusters are manually selected, the FIJI plugin calculates the pairwise 3D distances between the centers of mass of each spot per channel in all clusters. These distances were used in the DNA-FISH violin plots throughout this study. A Kolmogorov-Smirnov test was used to compare the distribution of distances between a given genotype and WT samples.

### ChIP-seq and C&T of insulator proteins in NC14 embryos

ChIP-seq was performed as described in (*79*). After sonication and chromatin extraction, the chromatin was aliquoted into fresh tubes and stored at −80°C until use. The quality of the sheared chromatin was determined by agarose gel electrophoresis to observe chromatin fragment size distribution. The following antibodies were used: rabbit anti-CTCF (gift from R. Reinkawitz), rat anti-CP190 (gift from P. Georgiev), goat anti-Su(Hw) (gift from P. Geyer), and rabbit anti-GAF (gift from J. Lis), which were incubated overnight with chromatin in radioimmunoprecipitation assay (RIPA) buffer [140 mM NaCl, 10 mM tris-HCl (pH 8.0), 1 mM EDTA, 1% Triton X-100, 0.1% SDS, 0.1% Na-deoxycholate, and 1× Roche cOmplete Protease inhibitors] in a total volume of 900 μl. We used 6 μg of chromatin for CTCF, 4 μg for CP190, 2 μg for Su(Hw), and 10 μg for GAF. Chromatin was fixed with 1.8% formaldehyde for 15 min for CTCF and Su(Hw) and with 3% formaldehyde for 30 min for CP190 and GAF. The next day, 25 μl of magnetic protein A/G beads (Dynabeads, Invitrogen, 10002D and 10004D) was washed with 1 ml of RIPA buffer and added to the samples for an additional 3-hour incubation on the rotating wheel at 4°C. For the BEAF-32 ChIP, 25 μl of protein G beads was combined with 100 μl of the BEAF-32 antibody (DSHB, #1553420) and 300 μl of RIPA buffer for 2 hours. This was followed by two washes with RIPA and resuspension in 100 μl of RIPA, which was added to the purified chromatin and incubated on the rotating wheel at 4°C overnight. The ChIPs were then washed for 10 min on the rotating wheel with 1× 1 ml of RIPA, 4× 1 ml of RIPA-500 [500 mM NaCl, 10 mM tris-HCl (pH 8.0), 1 mM EDTA, 1% Triton X-100, 0.1% SDS, 0.1% Na-deoxycholate, and 1× Roche cOmplete Protease inhibitors], 1× 1 ml of LiCl buffer [250 mM LiCl, 10 mM tris-HCl (pH 8.0), 1 mM EDTA, 0.5% IGEPAL CA-630 CA-630, and 0.5% Na-deoxycholate], and 2× 1 ml of TE buffer [10 mM tris (pH 8.0) and 1 mM EDTA] on a magnetic rack in the cold room. The chromatin was then ribonuclease-treated (#10109142001, Roche) and reverse cross-linked overnight with proteinase K (0.5 mg/ml) and 0.5% SDS at 65°C. The next day, the DNA was purified with phenol-chloroform purification and precipitated with ethanol, sodium acetate (pH 5.3), and glycogen to obtain pure DNA. Library preparation was performed using the NEBNext Ultra II DNA Library Prep Kit for Illumina [New England Biolabs (NEB), #E7645S], following the manufacturer’s instructions.

For quantitative C&T, spike-in with the same number of *D. virilis* nuclei was used. Nuclei were counted using the BD LSRFortessaTM X-20 flow cytometer at the EMBL Flow Cytometry Facility and snap-frozen. We thawed fixed *D. melanogaster* and *D. virilis* nuclei (50,000 each) and then mixed the nuclei from both species for a total of 100,000 nuclei per sample. A conA bead slurry was added to the sample containing both *D. melanogaster* and *D. virilis* nuclei and placed on a rotating wheel for 10 min at 4°C. The nuclei-bead complex was washed and permeabilized, and 1 μl of primary antibody was added to each sample. For the CTCF and CP190 C&T experiments, we used the same antibodies as listed for ChIP-seq experiments, and for the BEAF-32 C&T, we used a primary antibody gifted by C. Hart. The tubes were placed on a rotating wheel and slowly rotated (5 rpm) overnight at 4°C. A secondary antibody solution (1:100) was added to the beads and incubated on a nutator at room temperature for 1 hour. We used the following secondary antibodies: guinea pig anti-rabbit immunoglobulin G (IgG; H + L) (Antibodies-online, ABIN6923140), rabbit anti-rat IgG (H + L) (Thermo Fisher Scientific, A18917), and rabbit anti-mouse IgG (H + L) (Abcam, ab46540). Samples were then washed three times, and C&T was performed as described previously (*80*). The PCR products were purified with Agencourt AMPure XP beads (Beckman Coulter, #A63881), quantified with Qubit, and ran on the Bioanalyzer using hs DNA reagents and chips. Final libraries were multiplexed and sequenced with 75-bp paired-end reads using an Illumina NextSeq 500 platform at the EMBL Genomics Core Facility.

### Identifying disrupted TAD boundaries using Chromosight and Pareidolia

TAD boundary changes in 10-kb bin cooler matrices between WT and insulator-depleted samples were detected using Chromosight (*58*) and quantified with the Pareidolia tool (https://github.com/koszullab/pareidolia; release v1.2.0, accessed on 1 March 2022) (subsample = True, density_thresh = None, pearson_thresh = 0.0, and cnr_thresh = 0.0) using the 10-kb TAD boundaries as defined by HiCExplorer. Pareidolia quantifies the correlation of the Hi-C signal to the expected kernel (here, for TAD boundaries) and reports the difference (depletion minus WT, producing a negative score upon loss of correlation) together with a signal-to-noise ratio (snr) score that indicated how good was the separation between signal and noise in the evaluated submatrix. TAD boundaries with an snr of less than 5 were excluded. As Pareidolia does not provide any significance value on the reported differential score, we opted for a stratification approach where we compared the top changing boundaries (here, 100) to a set of 200 “stable” boundaries selected as the 200 boundaries with the smallest absolute differential score.

## Supplementary Materials

### This PDF file includes:

Figs. S1 to S4 References

## Notes

View/request a protocol for this paper from *Bio-protocol*.
