# Supplementary material for: CTCF, BEAF-32, and CP190 are not required for the establishment of TADs in early Drosophila embryos but have locus-specific roles
Source: Sci Adv. 2023 Feb 3;9(5):eade1085. doi: 10.1126/sciadv.ade1085 (PMC9897672; doi:10.1126/sciadv.ade1085)
Supplement: Supplementary file 1 — Figs. S1 to S4 References [file sciadv.ade1085_sm.pdf]

Science Advances
AAAS

Supplementary Materials for

CTCF, BEAF-32, and CP190 are not required for the establishment of TADs in early *Drosophila* embryos but have locus-specific roles

Gabriel R. Cavalheiro *et al.*

Corresponding author: Eileen E. M. Furlong, furlong@embl.de

Sci. Adv. **9**, eade1085 (2023)
DOI: 10.1126/sciadv.ade1085

The PDF file includes:

Figs. S1 to S4
Legends for tables S1 to S5
References

Other Supplementary Materials for this manuscript includes the following:

Tables S1 to S5

Supplementary Figures

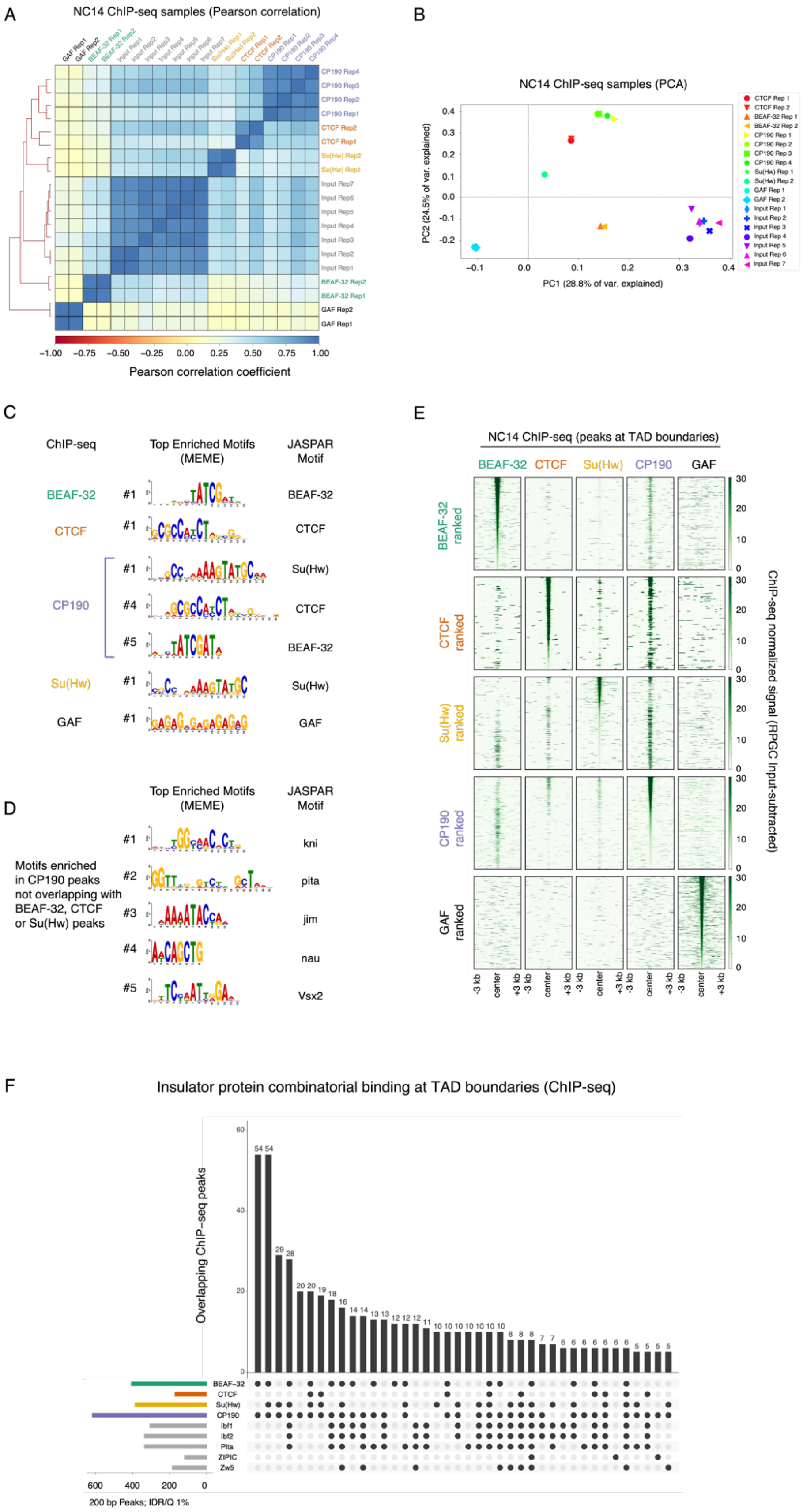

Supplementary Figure 1 (related to Figure 2): Properties of insulator ChIP-seq occupancy in early embryos. (A) Pearson correlation matrix comparing all unique, high-quality reads from all replicate and input insulator ChIP-seq experiments, showing that biological replicates cluster together, being most highly correlated to each other indicating the quality of the data. **(B)** Principle Component Analysis (PCA) of all ChIP-seq replicates and inputs, showing clear separation between factors. **(C)** Top enriched transcription factor motifs (using MEME) within the ChIP peaks for each insulator. The BEAF-32, CTCF, Su(Hw) and GAF motifs are the top most highly enriched motif (#1) in their respectively ChIP peaks. CP190 peaks co-bound with other insulator proteins have top enriched motifs (#1, #4 and #5) for Su(Hw), CTCF and BEAF-32. **(D)** CP190 peaks not co-bound with the other insulators tested here top enriched motifs (using MEME), identifies motifs for other potential recruiters. **(E)** Heatmap of insulator proteins normalized ChIP signal (reads per genome coverage (RPGC) input subtracted) at TAD boundaries (10kb resolution), centered at ChIP-seq peak summits. Each of the 5 rows of plots are ranked by the signal intensity of the ChIP-seq for the insulator indicated. **(F)** UpSet plot of combinatorial insulator protein binding at TAD boundaries (summits within the 10 kb boundary region). Binding data for the factors listed in grey (Ibf1 (*46*), Ibf2 (*46*), Pita (*47*), ZIPIC (*47*) and Zw5 (*47*)) was obtained from studies performed either in cell culture or at later embryonic stages.

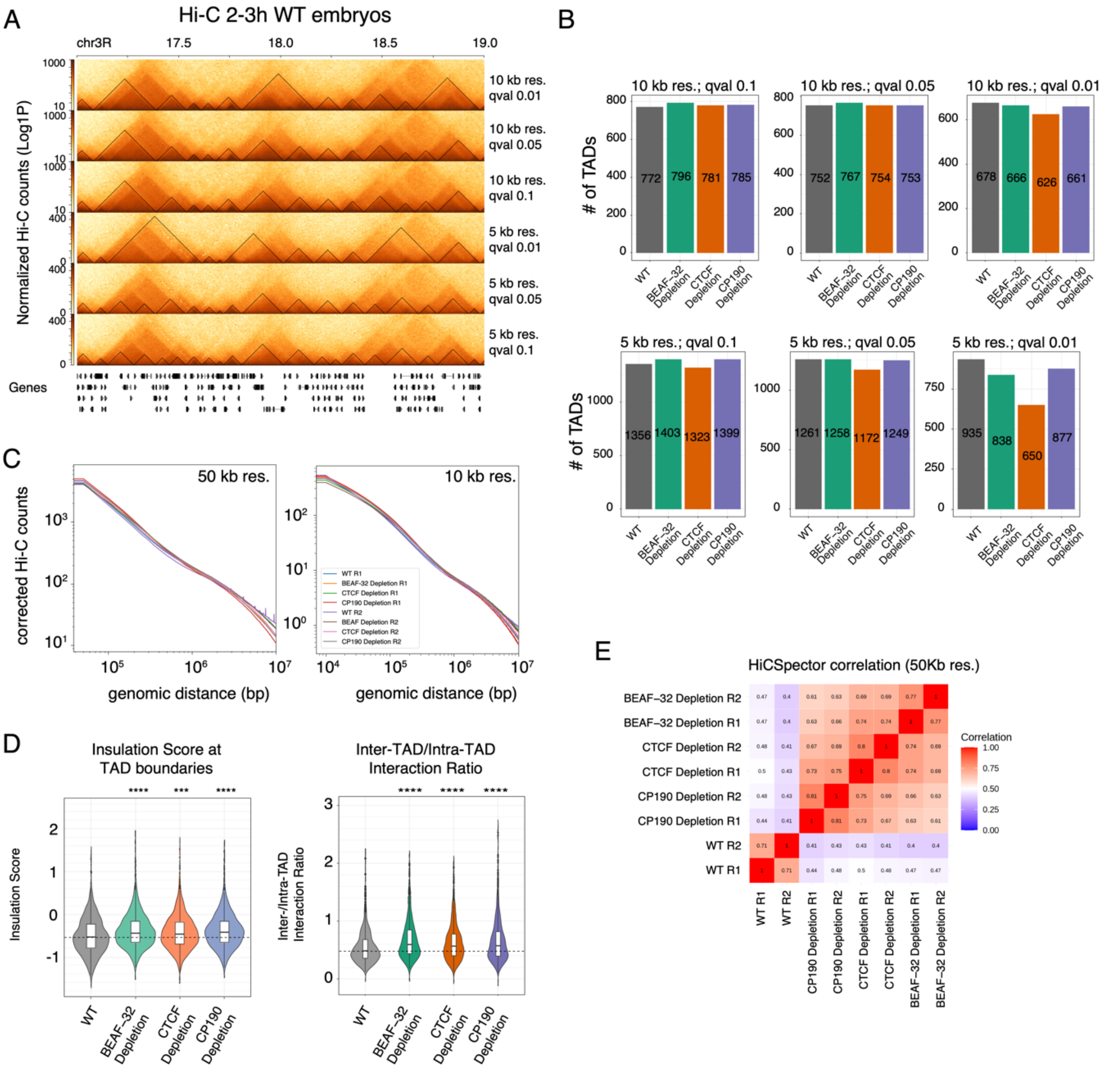

Supplementary Figure 2 (related to Figure 4): TAD calls using different parameters and resolution. (A) Hi-C matrices from 2-3h WT embryos, comparing the same genomic region across two different resolutions (10kb and 5kb) and 3 different q-values (0.1, 0.05 and 0.01; used for multiple testing correction when calling boundaries ((*23*) and see Methods). The 10kb resolution consistently gave TAD calls that better represent the visual inspection of TADs, and avoids the division of large domains into smaller sub-domains. **(B)** Quantification of the number of TADs called across the conditions used in (A). **(C)** Hi-C counts along genomic distances for each Hi-C replicate at 50kb and 10kb resolutions, indicating no major differences in the distributions of contacts along scales between genotypes. **(D)** *Left:* Quantification of insulation score at all TAD boundaries in WT vs. insulator depleted embryos. *Right:* Quantifications of the ratio between intra- vs. inter-TAD Hi-C contacts across the genome in WT vs insulator depleted embryos (2-3h). Values below 1.0 indicate higher intra-TAD contacts, while increases in this ratio indicate increase in interactions across boundaries (inter-TAD), as observed in the insulator depletions. Kolmogorov–Smirnov (KS) test, **** $p < 0.0001$. **(E)** HiCSpector correlation between Hi-C replicates measured at 50kb resolution, indicating that replicates for all genotypes cluster together, and all insulator depletions cluster together separately from the WT replicates.

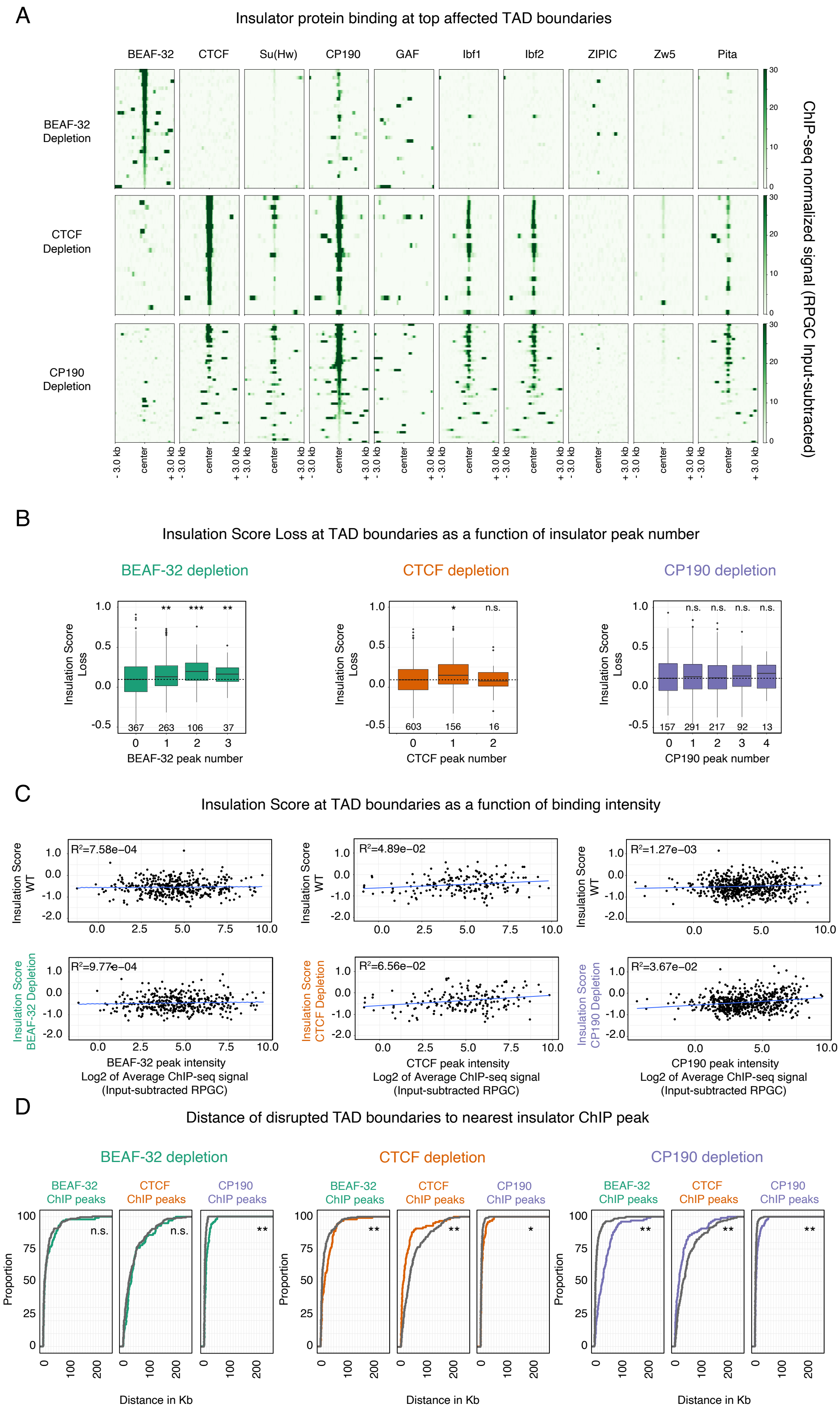
A
Insulator protein binding at top affected TAD boundaries
BEAF-32
CTCF
Su(Hw)
CP190
GAF
Ibf1
Ibf2
ZIPIC
Zw5
Pita
BEAF-32 Depletion
CTCF Depletion
CP190 Depletion
- 3.0 kb
center
+ 3.0 kb
ChIP-seq normalized signal (RPGC Input-subtracted)
B
Insulation Score Loss at TAD boundaries as a function of insulator peak number
BEAF-32 depletion
CTCF depletion
CP190 depletion
Insulation Score Loss
BEAF-32 peak number
CTCF peak number
CP190 peak number
C
Insulation Score at TAD boundaries as a function of binding intensity
Insulation Score WT
R²=7.58e-04
R²=4.89e-02
R²=1.27e-03
Insulation Score BEAF-32 Depletion
Insulation Score CTCF Depletion
Insulation Score CP190 Depletion
R²=9.77e-04
R²=6.56e-02
R²=3.67e-02
BEAF-32 peak intensity
CTCF peak intensity
CP190 peak intensity
Log2 of Average ChIP-seq signal
(Input-subtracted RPGC)
D
Distance of disrupted TAD boundaries to nearest insulator ChIP peak
BEAF-32 depletion
CTCF depletion
CP190 depletion
BEAF-32 ChIP peaks
CTCF ChIP peaks
CP190 ChIP peaks
Proportion
Distance in Kb

Supplementary Figure 3 (related to Figure 4): The distance of an insulator bound region to a disrupted TAD boundary. **(A)** Heatmap of insulator protein ChIP-seq normalized signal (reads per genome coverage (RPGC) input subtracted), centered at ChIP-seq peak summits. Each set (row) displays ChIP-seq signal intensity of the indicated insulator proteins in WT embryos, at the top-affected TAD boundaries in a given genotype. **(B)** Distribution of insulation score loss at TAD boundaries in BEAF-32, CTCF and CP190 depleted embryos (left, middle and right panels) compared to WT embryos, at boundaries occupied by increasing number of insulator peaks (in WT embryos). Kolmogorov–Smirnov (KS), two-sided test. n.s. ($p > 0.05$), * ($p < 0.05$), ** ($p < 0.01$), *** ($p < 0.001$). **(C)** Distribution of insulation score at TAD boundaries in WT embryos (top) and BEAF-32, CTCF and CP190 depleted embryos (bottom), as a function of the ChIP signal intensity (in WT embryos). **(D)** Cumulative curves of the distance between disrupted TAD boundaries in each depletion (colored lines) to the nearest insulator ChIP-seq peak (in WT embryos). As a control, the same distances for a random 100 non-affected boundaries are shown (grey lines). Distances are computed from the center position within the 10kb boundary bin. Kolmogorov–Smirnov (KS), two-sided test. n.s. ($p > 0.05$), * ($p < 0.05$), ** ($p < 0.01$).

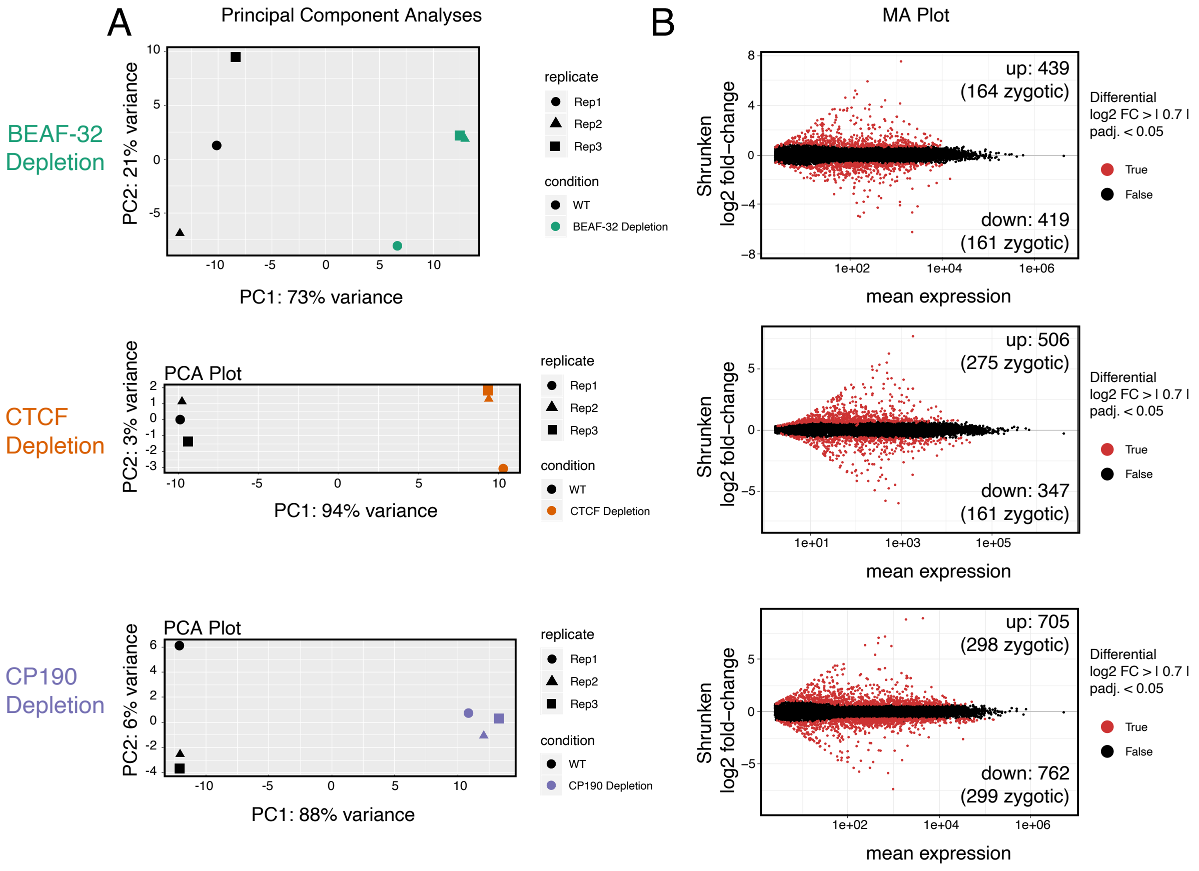

Supplementary Figure 4 (related to Figure 5): Changes in gene expression in NC14 embryos after insulator protein depletion. (A) Principle Component Analysis (PCA) of all three RNA-seq replicates (normalised read counts) from WT and insulator depleted embryos, showing clear separation between the WT (black) and insulator depleted (coloured) samples. **(B)** MA plot, using shrunken log2 fold change (FC) (y-axis) and mean expression (x-axis) of RNA-seq in WT versus insulator depleted embryos at NC14. Genes with a | log2 FC | > 0.7 and FDR < 0.05 indicated in red.

Supplementary Methods

Western Blots

For each sample, we manually selected 100 NC14 *Drosophila* embryos and directly placed them in sample buffer. Embryos were lysed manually with a plastic pestle (#12-141-363, Thermo Fisher Scientific) in a protein low-binding tube (#90410, Thermo Fisher Scientific) and sample buffer was added for a total of 100 µl. The embryo extract was incubated at 95°C for 10 minutes. This was followed by maximum speed centrifugation, after which the supernatant was transferred to a new protein low-binding tube and stored at -80°C. All Western blots were performed with biological triplicate samples. Protein samples were heated at 90°C for 10 minutes, and 25 µl loaded on a 4-20% gradient acrylamide gel (Mini-PROTEAN® TGX Stain-Free) (#4568095, Bio-Rad) and ran with 1x laemmli running buffer in a vertical electrophoresis system (#1658004, Bio-Rad). Proteins were transferred to a Nitrocellulose membrane (#GE10600002, Amersham) at 80V for 1h15 at 4°C, using the Mini Trans-Blot® Cell system (#1703930, Bio-Rad), following manufacturer's recommendations. Protein transfer was assessed by Ponceau staining, followed by washes with dH2O and with PBS + 0.1% Tween. The membrane was blocked with 5% milk for 1h and incubated with primary antibodies diluted as follows in PBS with 0.1%Tween, 5% milk: anti-CTCF (1:5000, gift from R. Reinkawitz), anti-CP190 (1:5000, gift from P. Georgiev) , anti-BEAF-32 (1:150, DSHB) or anti-alpha-Tubulin (1:10000, Abcam #ab7291). After removing the solution with the primary antibody (which was stored at 4°C for subsequent use) the membrane was washed 3x with PBS + 0.1% Tween and incubated with an appropriate secondary antibody diluted 1:10000 in PBS + 0.1% Tween, 5% milk for 2 hours at room temperature. Membranes were developed using SuperSignal West Pico Chemiluminescent Substrate (#34079, Thermo Fisher Scientific) in a ChemiDoc MP Imaging System (#17001402, Bio-Rad). Western blots were quantified using ImageJ, via its distribution FIJI (*82*). In each experiment, the mean value of three alpha-Tubulin bands was calculated and used to normalise loading differences. The signal of each insulator protein band was divided by the normalizer, and values were corrected for background intensity. A t-test was performed to determine statistical significance.

Double fluorescent in-situ hybridization of gene expression

Whole-mount *Drosophila* RNA *in-situ* hybridisation experiments were carried out as described previously (*83*). RNA *in situ* anti-sense probes were prepared from corresponding

EST clones obtained from DGRC for *btsz* (LP02621), *wg* (RE02607), *wnt4* (RE26454), *sog* (LP09189) and *CG12708/CG15646* (LD21346) using Digoxigenin-, Biotin- or Fluorescein-modified nucleotides. mRNA expression was visualized from these probes using anti-Digoxigenin-Peroxidase (Roche #11633716001), anti-Biotin-Peroxidase (Sigma-Aldrich #A4541) and anti-Fluorescein-Peroxidase (Roche #11426346910) (all antibodies diluted 1:2000) coupled with the TSA Plus Fluorescence kit (PerkinElmer #NEL760001KT). Stained embryos were mounted in ProLong™ Gold mounting medium with DAPI (ThermoFisher Scientific #P36931) and imaged on a Leica SP8 confocal laser-scanning microscope with an HC Pl APO CS2 20x/0.75 IMM.

RNA-seq

RNA-seq experiments were performed in biological triplicates per genotype. For each replicate, 100 embryos at NC14 were manually selected, following a 2-3h collection. Embryos were dechorionated, air-dried, snap-frozen in liquid nitrogen and stored at -80C until use. Embryos were homogenized in TRIzol LS (#10296028, ThermoFisher Scientific) with a Cordless Motor for Pellet Mix and pestles (#47747-370, VWR) on ice. RNA was extracted according to the manufacturer's instructions, and the remaining DNA was digested with RNase-free DNase I (#4716728001, Roche) for 30 min. Strand-specific RNA-seq was performed from 1 μg of total RNA using the NEBNext Ultra Directional RNA Library Prep Kit for Illumina (#E7420, NEB) according to the manufacturers' instructions. For each genotype, RNA-seq was performed in three biological replicates, representing three independent embryo collections. Final libraries were ran on a Bioanalyzer (Agilent), multiplexed and sequenced on an Illumina NextSeq 500 (75-bp paired end) platform at the EMBL Genomics Core Facility

For **RNA-seq read processing** the sequencing adapter sequences were removed from the 3' end of reads using Cutadapt (version 1.18 (*84*)), removing the adapter sequence AGATCGGAAGAGC. Reads were aligned to the Dm6 genome using STAR (version 2.6.1b (*85*)). The STAR genome index was generated using a Dm6.13 GTF file. Gene counts were generated using RSEM (version 1.3.1 (*86*)) and any non-integer counts were rounded to the nearest whole number. Differential analysis was performed using DESeq2 (version 1.22.1 (*87*)) with the use of independent filtering and an alpha of 0.05. Differential genes were defined as those with an adjusted p-value of < 0.05 and absolute log2 fold-change >= 0.7. When plotting log2 fold-change values, the normal shrunken log2 fold-change values

as generated by DESeq2 were used. Maternally deposited genes were defined as genes expressed in unfertilized eggs, using the *vgn* line of *Drosophila melanogaster* at 2–4 or 6–8 h after egg laying (*10*). For all analyses, maternal genes were removed, and only zygotic genes were kept.

ChIP-seq data processing and peak calling

Primary processing of the ChIP-Seq data was performed in the Galaxy platform using a workflow composed of the main following steps:

1. Read cleaning using *Trim Galore! (Galaxy Version 0.4.3.1)* with automatic adaptor detection, Trim low-quality ends from threshold: 20, Overlap with adapter sequence required to trim a sequence: 2, Maximum allowed error rate: 0.1, reads becoming shorter than 20 were discarded.
2. Read Mapping to the dm6 genome using *Bowtie2 (Galaxy Version 2.3.4.2)* with paired-end options and --sensitive -I 0 -X 2000 --fr --dovetail (all other options left to default)
3. Read filtering using *Filter SAM or BAM, output SAM or BAM files on FLAG MAPQ RG LN or by region (Galaxy Version 1.1.2)* to only keep read mapped in a proper pair with MAPQ > 19 (which eliminates multi-mapping reads). We also only kept read pairs that mapped to major chromosomes (chr2L, chr2R, chr3L, chr3R, chr4 and chrX)
4. Read deduplication using *Picard MarkDuplicates (Galaxy Version 2.7.1.1)*
5. "10bp 1X-normalized" BigWig Signal Files were created for each IP and input samples using *DeepTools bamCoverage (Galaxy Version 3.0.2.0)* at a 10bp resolution with the normalized to 1x coverage option.
6. Input subtracted BigWig Signal Files were created for each IP sample by subtracting the input signal from the IP signal using *DeepTools bamCoverage (Galaxy Version 3.0.2.0)* at a 10bp resolution
7. ChIP-seq quality was assessed using different tools:
   a. *FastQC (Galaxy Version 0.69)* at different steps of the workflow to check sequencing quality and monitor filtering step efficiency
   b. *Picard Collect Alignment Summary Metrics (Galaxy Version 2.7.1.1)* to evaluate library quality across samples (read duplication and unmapped reads rates)

c. *Picard CollectInsertSizeMetrics (Galaxy Version 2.7.1.0)* to compare fragment length
d. *Deeptools plotFingerprint (Galaxy Version 3.0.2.0)* to check ChIP signal strength

8. PCA and correlation matrices using all IP and input sample replicates were generated with *Deeptools multiBigwigSummary (Galaxy Version 3.0.2.0)* using 10bp 1X normalized BigWig files followed by *plotCorrelation (Galaxy Version 3.0.2.0)* and *plotPCA (Galaxy Version 3.0.2.0)* and used to assess replicates' reproducibility

Peak Calling incorporated IDR analysis, which was performed on pairs of replicates with default parameters using *IDR (Galaxy Version 2.0.3)* on 100 bp ChIP regions centered on the summits obtained using *MACS2 callpeak (Galaxy Version 2.1.1.20160309.5)* performed on the filtered IP BAM files (uniquely mapped reads) with the following options: --call-summits, a relaxed p-value cutoff of 0.5 (as required by IDR analysis), matched input BAM as control and default paired-end options. Only regions with IDR below 1% were further considered. When more than two replicates were available (4 replicates in the CP190 case), we ran all pairwise comparisons (6 IDR runs in CP190 case). The peaks from all 6 IDR runs with an IDR less or equal to 0.01 were merged by overlap with *bedtools merge v2.27.1*. To avoid a potential bias of the IDR region width distribution (CP190 regions are potentially larger due to the merging between regions coming from 6 pairwise comparisons), we post-processed all the IDR peaks with the galaxy workflow:

1. Call peaks using all replicates (2 or 4 in CP190 case) using *MACS2 callpeak (Galaxy Version 2.1.1.20160309.5)* on the filtered IP BAM files (uniquely mapped reads) with the following options: --call-summits, p-value cutoff of 0.5 (similar as for the IDR analysis), matched input BAM as control and default paired-end options.
2. All MACS peak summits not falling in IDR 1% regions were discarded (Intersect intervals Galaxy Version 2.27.0.2)
3. Each remaining 1bp summit position is then slopped by 100 bp (resulting in 200 bp regions centered on the summit) with *SlopBed Galaxy Version 2.27.0.0*
4. *MergeBED Galaxy Version 2.27.0.0* is finally applied to remove potentially overlapping regions, followed by *SortBED Galaxy Version 2.27.0.0* to sort final regions

Whenever applicable, we used the matched input as a control. In a number of cases, the input had much more reads than its IP counterpart and we adjusted the input read number by

random subsampling (*Samtools view reformat, filter, or subsample, Galaxy Version 1.9*). The regions resulting from this workflow are the final "IDR 1% ChIP peaks" used in this paper.

Bigwig signal files for each condition (e.g. CTCF at stage NC14), were generated by making merged input-subtracted signal files as follows: IP and matched input replicate signal files (10bp 1X-normalized bigwig, see above) were summed up (IP and input separately) and the summed input signal was subtracted from the summed IP signal using *DeepTools bamCoverage (Galaxy Version 3.0.2.0).* These files are used across the figures i.e. heatmaps (*DeepTools computeMatrix* followed by *DeepTools plotHeatmap*) and for visualisation of genomic regions (*pyGenomeTracks 3.5.1*).

To **assess combinatorial binding,** all final "IDR 1% ChIP peaks" were merged by overlap with *bedtools merge v2.27.1* into a unique set of non-overlapping "Insulator Binding Regions". For each of these Insulator Binding Regions, we computed (1) the list of binding insulators by intersecting with the original IDR 1% ChIP peaks (*bedtools intersect v2.27.1*) and (2) the average binding signal of each insulator with *Deeptools multiBigwigSummary 3.1.3* using the merged signal BigWig files (see above). UpSet plots were generated using the R UpSetR package. Motif discovery was performed using MEME-chip 5.3.0 on the 200 bp "IDR 1% ChIP peaks" defined above, using default options.

Hi-C

For nuclei isolation, 1.8% formaldehyde fixed embryos stored at -80°C were thawed in 10 ml ice cold HB buffer (15 mM Tris-HCl pH 7.4, 15 mM NaCl, 60 mM KCl, 340 mM sucrose, 0.2 mM EDTA pH 8, 0.2 mM EGTA pH 8, 1x Roche cOmplete Protease inhibitors) in a 15 ml tissue homogeniser (Wheaton). The embryos were dounced 20x with the loose glass-on-glass Wheaton pestle and 20x with the tight pestle. The homogenised material was filtered through 2 layers of miracloth (Calbiochem, #475855-1R) and spun at 3200g for 10 min at 4°C. The pellet was resuspended in 10 ml HB buffer and centrifuged at 3200g for 10 min at 4°C. The nuclei were then resuspended in 3 ml of PBS + 0.1% Triton + 1x Roche cOmplete Protease inhibitors. In order to obtain single nuclei for counting, the suspension was passed ten times through a 20G and ten times through a 22G needle and filtered through 20 μm Nitex membrane (Sefar Nitex Switzerland, #03-20/14). Following nuclei counting using the BD LSRFortessaTM X-20 Flow Cytometer at the EMBL Flow Cytometry Facility, aliquots of 15 million nuclei were stored at -80°C until use. We used a "Bridge-Adaptor *in situ* Hi-C

protocol", corresponding to an in situ Hi-C protocol (*14*) utilizing biotinylated bridge oligo adaptors to allow for multiplexing between samples, as described in (*88*). Hi-C experiments were performed in biological duplicates per genotype.

Prior to the Hi-C experiment, biotinylated bridge oligos were prepared as follows:

10 µl 10X NEBuffer 2 was mixed with 90 µl of a 100 µM mixture of the two oligos (Bridge oligo forward: GATCGAGCTCGAGAA/iBiodT/T, Bridge oligo reverse: CTCGAGCTC). The mixture was heated to 98°C for 6 min, then ramped down to RT at -0.1°C / sec and stored at -20°C until use.

15 million nuclei were thawed on ice and 1 ml of lysis buffer (10 mM Tris-Cl pH 8.0, 10 mM NaCl, 0.2% IGEPAL CA-630, 1x cOmplete protease inhibitor (Roche, Cat. No. 11 873 580 001)) was added. Samples were incubated on ice for 30 min and then spun at 1000xg at 4°C for 5 min. The supernatant was removed, pellets were resuspended in 200 µl of 0.5% SDS and incubated at 65°C for 10 min. The SDS was then quenched by adding 100 µl of 10% Triton X-100 and incubating at 37°C for 15 min.

50 µl of 10x NEBuffer 3.1, 130 µl nuclease-free H2O and 8 µl of DpnII (NEB) were added and samples were incubated at 37°C overnight. Tubes were centrifuged for 5 min at 1000xg at RT and pellets were resuspended in 30 µl nuclease-free H2O. 5 µl Biotinylated bridge oligos (90 µM) were ligated to the DNA fragments overnight at 16°C using 1 µl of T4 DNA ligase HC (Thermo Fisher), 5 µl ligation buffer, 4 µl 10x BSA, and 5 µl PEG 4000. After adding 2.5 µl of 0.5 M EDTA, the samples were centrifuged at 1000xg at RT for 5 min and resuspended in 300 µl 1x BSA and 0.2% SDS in water. The pellets were centrifuged again at 1000xg at RT for 5 min and the pellets were resuspended in 300 µl 1x BSA and 0.1% SDS in water. This step was repeated for a second time. After another centrifugation, the samples were resuspended in 245 µl of 1.22x BSA and 0.2% Triton X-100 in water.

The bridge oligos were phosphorylated by adding 30 µl 10x T4 ligase buffer (Thermo Fisher) and 20 µl PNK (10 U/ µl, NEB). Tubes were incubated at 37°C for 1h. Bridge oligos were ligated to each other using 5 µl T4 DNA ligase HC (Thermo Fisher), 70 µl 10x T4 ligase buffer, 7 µl 100x BSA and 618 µl nuclease-free H2O. Samples were incubated for 4 h at RT. The samples were then spun down at 1000xg at 4°C for 5 min and the pellet was resuspended in 500 µL of extraction buffer (50 mM Tris-Cl pH 8.0, 50 mM NaCl, 1 mM EDTA, 1% SDS).

To digest proteins, 20 µL of 20 mg/ml Proteinase K (Invitrogen) was added and the mixture was incubated at 55°C at 1000 rpm on a thermal shaker for 30 min. For decrosslinking 130 µL of 5 M sodium chloride were added and the mixture was incubated at

68°C at 1000 on a thermal shaker overnight. To precipitate DNA, 63 μL of 3 M sodium acetate pH 5.2, 2 μL of 15 mg/ml GlycoBlue (Thermo Fisher) and 1000 μL of absolute ethanol were mixed with the sample, followed by incubation at −80°C for 1 hour. The samples were then spun at 20,000xg at 4°C for one hour. The supernatant was removed and the DNA pellet washed two times using 800 μL of 70% ethanol. All traces of remaining supernatant were removed; the pellet was air-dried for 2 min and then solubilized in 99 μl of 10 mM Tris-Cl pH 8.0. RNA was digested by adding 1 μL of 10 mg/ml RNase A and incubated at 37°C for 15 min. The DNA was sheared in a total volume of 100μl to ~200-400 bp with Diagenode Bioruptor Pico (4°C, ON/OFF 30''/90'', 8 cycles). DNA fragments were size-selected with SPRIselect Beads (Beckman Coulter) following the manufacturer's instructions. For right side size selection 0.6x volume of beads were used, for left side size selection 1x was used. DNA was eluted in 100μL of 10mM Tris-Cl pH 8.0.

For biotin enrichment, 30 μl of Dynabeads Streptavidin M-280 (Life Technologies) per sample were prepared following the manufacturer's instructions and added to the purified DNA. The biotinylated bridge oligos were allowed to bind to the beads for 20 min at RT. The beads were washed 4 times with 200 μL each of 1x B&W buffer (5 mM Tris-HCl pH 7.5, 0.5 mM EDTA, 1 M NaCl) + 0.1% Tween-20. This was followed by 2 washes with 10 mM Tris-Cl pH 8.0. The beads were then resuspended in 40 μL of the same solution.

Library preparation was done using Swift Biosciences Accel-NGS 2S Plus DNA Library Kit, with the following deviation from the manufacturer's manual: throughout library preparation no SPRIselect clean-up was performed. Instead, the samples were washed using the already bound Streptavidin beads. Each of the SPRI Steps of the Swift Biosciences Accel-NGS 2S Plus DNA Library Kit manual was replaced by the washes described hereafter: Samples were placed against a magnet, and incubated until the solution is clear. The supernatant was discarded and samples were removed from the magnet. Beads were resuspended in 150 μL of 1x B&W buffer + 0.1% Tween-20, and incubated at 55°C in a thermal cycler for 2 min. This wash step was repeated one more time. An additional wash step with 100μL of 10 mM Tris-Cl pH 8.0 was performed. H ere, the samples were immediately put against the magnet after resuspension, without any incubation. The supernatant was discarded and samples were removed from magnet. Beads were resuspended in the mixes as described in the Swift Biosciences Accel-NGS 2S Plus DNA Library Kit manual for Repair II, Ligation I and Ligation II.

After Ligation II and the above described washes, beads were resuspended in 20µL of 10 mM Tris-Cl pH 8.0. 25 µl HiFi HotStart ReadyMix and 5 µl 10x Primer Mix (both Kapa Biosystems) were added and the PCR program as described in the Kapa HiFi HotStart ReadyMix manual was run on the samples. We used 60°C annealing temperature, 30 sec extension time and 12 cycles for the amplification. After the PCR, two 0.9x SPRI select clean-ups were made and library concentration was measured using the Qubit dsDNA HS Assay kit (Thermo Fisher). Fragment size of the libraries was determined with High Sensitivity DNA Kit for Bioanalyzer (Agilent Technologies). Libraries were sequenced using standard Illumina reagents with a read length of 150 bp, paired-end.

Hi-C data processing and TAD calls

Hi-C data was processed using the HiCExplorer suite version 3.6 unless otherwise stated. Two biological replicates were sequenced for each condition (WT, CTCF Depletion, BEAF-32 Depletion and CP190 Depletion) in two different lanes thus generating two different technical replicates for each biological replicate. A first galaxy workflow was assembled to compute the raw contact matrices for each biological replicate separately (i.e. merging all technical replicates together after mapping):

1. Mapping forward and reverse reads separately to the dm6 genome using *Map with BWA-MEM* (Galaxy Version 0.7.17.1) with default parameters but -E 50 -L 0 -5
2. Merging the technical replicate BAM files together with *MergeSamFiles* (Galaxy Version 2.18.2.1)
3. Sort reads by name using *Samtools sort* (Galaxy Version 2.0.2)
4. Compute raw contact matrix at 1K resolution using *hicBuildMatrix* (Galaxy Version 3.6+galaxy0) with options: GATC as the restriction site and dangling sequence, –minMappingQuality 10 and the list of restriction fragments as produced by *hicFindRestSite* (Galaxy Version 3.6+galaxy0) ran on the dm6 FASTA genome with --searchPattern GATC.
5. The trans-contacts were filtered out using *hicAdjustMatrix* version 3.7 with options -a keep --interIntraHandling inter --chromosomes chr2L chr2R chr3L chr3R chr4 chrX

The table below lists the total number of cis-contacts for each replicate and indicates a good read number balancing and no sequencing depth bias. Given those, we judged that no subsampling was required before further processing.

Replicate	Usable Reads (% of the mapped reads)	Cis-interaction count	Merged
BEAF_RNAi_2-3h_rep1	65%	103,692,660	197,351,729
BEAF_RNAi_2-3h_rep2	66%	93,659,069	
CP190_RNAi_2-3h_rep1	74%	113,377,887	195,967,147
CP190_RNAi_2-3h_rep2	60%	82,589,260	
CTCF_KO_2-3h_rep1	50%	78,879,585	169,184,598
CTCF_KO_2-3h_rep2	56%	90,305,013	
WT_2-3h_rep1	83%	127,601,768	228,878,289
WT_2-3h_rep2	83%	101,276,521	

Further processing included the generation of raw contact matrices of coarser resolutions (5K, 10K…) using *hicMergeMatrixBins* and merging biological replicates raw matrices using *hicSumMatrices*. The different raw contact matrices (at different resolutions, replicate-merged or not) were normalized using *hicCorrectMatrix* (ICE/Imakaev's iterative correction). Note that we always executed *hicCorrectMatrix* in diagnostic mode first to extract optimal min/max values to pass to the –filterThreshold option. We used *hicCompareMatrices* 3.7.2 to generate log2ratio interaction matrices from the ICE matrices with options --noNorm --operation log2ratio. Genomic regions views were generated with *pyGenomeTracks 3.5.1*.

Hi-C quality control was assessed using replicate reproducibility using the 3DChromatin_ReplicateQC tool; in particular we show the results from the HiCSpector module gained on normalized 50 Kb binned matrices (Supplementary Fig. 3). Quality of the different samples was evaluated by checking the percentage of mapped reads (around 90% for all replicates), the percentages of self-ligation, same-fragment, self-circle and duplicates as reported by the hicBuildMatrix steps. The final fraction of usable reads (relative to the properly mapped reads) is indicated in the table above. The different proportions of usable reads are due to the different proportions of duplicates. Finally, we observed different proportions of inter-chromosomal contacts for BEAF-32 depletion, CP190 depletion and CTCF depletion (25-30%) and the WT 2-3h (35-40%); which we decided to normalize out by excluding trans contacts from the beginning of the normalization process.

TAD calls and Insulation Score: TADs were called on normalized merged matrices using *hicFindTADs* with three different q-value cut-offs (0.01, 0.05 and 0.1) on 5K and 10K binned matrices. Following the authors recommendations, we set --minDepth 15000 –maxDepth 50000 and --step 5000 for the 5K binned matrices and --minDepth 30000 –maxDepth 100000 and --step 10000 for the 10K binned matrices. The final TAD calling parameters were set to 10K with a q-value threshold of 0.1 after extensive manually comparison of the different TAD call sets. Of note, the insulation score used in the different analysis is that directly provided by *hicFindTADs.* The insulation score loss was defined as the difference between the mutant and the wild type; a positive insulation score loss therefore reflects a lower insulation score in the mutant.

The following regions close to chromosome ends showed aberrant HiC profiles and were therefore excluded from analysis involving HiC data. The boundaries were defined upon manual inspection of the WT HiC maps: chr2L:21000000-23513712; chr2R:0-6000000; chr3L:23000000-28110227; chr3R:0-4000000; chrX:21400000-23542271; chr4:0-1348131

Supplementary Data

Table S1: List of TAD boundaries affected by insulator protein depletions

Table S2: List of Differentially Expressed Genes (DEGs) upon insulator protein depletions

Table S3: RNA-seq changes in genes activated during the minor ZGA and involved in early patterning upon insulator protein depletions

Table S4: List of top 100 affected boundaries in each genotype, and potential enhancer hijacking cases

Table S5: DNA oligos used in this study

REFERENCES AND NOTES

1. J. Dekker, E. Heard, Structural and functional diversity of topologically associating domains. *FEBS Lett.* **589**, 2877–2884 (2015).

2. J. R. Dixon, S. Selvaraj, F. Yue, A. Kim, Y. Li, Y. Shen, M. Hu, J. S. Liu, B. Ren, Topological domains in mammalian genomes identified by analysis of chromatin interactions. *Nature* **485**, 376–380 (2012).

3. E. P. Nora, B. R. Lajoie, E. G. Schulz, L. Giorgetti, I. Okamoto, N. Servant, T. Piolot, N. L. van Berkum, J. Meisig, J. Sedat, J. Gribnau, E. Barillot, N. Bluthgen, J. Dekker, E. Heard, Spatial partitioning of the regulatory landscape of the X-inactivation centre. *Nature* **485**, 381–385 (2012).

4. T. Sexton, E. Yaffe, E. Kenigsberg, F. Bantignies, B. Leblanc, M. Hoichman, H. Parrinello, A. Tanay, G. Cavalli, Three-dimensional folding and functional organization principles of the Drosophila genome. *Cell* **148**, 458–472 (2012).

5. B. Bintu, L. J. Mateo, J. H. Su, N. A. Sinnott-Armstrong, M. Parker, S. Kinrot, K. Yamaya, A. N. Boettiger, X. Zhuang, Super-resolution chromatin tracing reveals domains and cooperative interactions in single cells. *Science* **362**, eaau1783 (2018).

6. L. Giorgetti, R. Galupa, E. P. Nora, T. Piolot, F. Lam, J. Dekker, G. Tiana, E. Heard, Predictive polymer modeling reveals coupled fluctuations in chromosome conformation and transcription. *Cell* **157**, 950–963 (2014).

7. O. Symmons, V. V. Uslu, T. Tsujimura, S. Ruf, S. Nassari, W. Schwarzer, L. Ettwiller, F. Spitz, Functional and topological characteristics of mammalian regulatory domains. *Genome Res.* **24**, 390–400 (2014).

8. K. C. Akdemir, V. T. Le, S. Chandran, Y. Li, R. G. Verhaak, R. Beroukhim, P. J. Campbell, L. Chin, J. R. Dixon, P. A. Futreal, PCAWG Structural Variation Working Group; PCAWG Consortium, Disruption of chromatin folding domains by somatic genomic rearrangements in human cancer. *Nat. Genet.* **52**, 294–305 (2020).

9. W. A. Flavahan, Y. Drier, B. B. Liau, S. M. Gillespie, A. S. Venteicher, A. O. Stemmer-Rachamimov, M. L. Suva, B. E. Bernstein, Insulator dysfunction and oncogene activation in IDH mutant gliomas. *Nature* **529**, 110–114 (2016).

10. Y. Ghavi-Helm, A. Jankowski, S. Meiers, R. R. Viales, J. O. Korbel, E. E. M. Furlong, Highly rearranged chromosomes reveal uncoupling between genome topology and gene expression. *Nat. Genet.* **51**, 1272–1282 (2019).

11. D. G. Lupianez, K. Kraft, V. Heinrich, P. Krawitz, F. Brancati, E. Klopocki, D. Horn, H. Kayserili, J. M. Opitz, R. Laxova, F. Santos-Simarro, B. Gilbert-Dussardier, L. Wittler, M. Borschiwer, S. A. Haas, M. Osterwalder, M. Franke, B. Timmermann, J. Hecht, M. Spielmann, A. Visel, S. Mundlos, Disruptions of topological chromatin domains cause pathogenic rewiring of gene-enhancer interactions. *Cell* **161**, 1012–1025 (2015).

12. A. Despang, R. Schopflin, M. Franke, S. Ali, I. Jerkovic, C. Paliou, W. L. Chan, B. Timmermann, L. Wittler, M. Vingron, S. Mundlos, D. M. Ibrahim, Functional dissection of the Sox9-Kcnj2 locus identifies nonessential and instructive roles of TAD architecture. *Nat. Genet.* **51**, 1263–1271 (2019).

13. E. de Wit, E. S. Vos, S. J. Holwerda, C. Valdes-Quezada, M. J. Verstegen, H. Teunissen, E. Splinter, P. J. Wijchers, P. H. Krijger, W. de Laat, CTCF binding polarity determines chromatin looping. *Mol. Cell* **60**, 676–684 (2015).

14. S. S. Rao, M. H. Huntley, N. C. Durand, E. K. Stamenova, I. D. Bochkov, J. T. Robinson, A. L. Sanborn, I. Machol, A. D. Omer, E. S. Lander, E. L. Aiden, A 3D map of the human genome at kilobase resolution reveals principles of chromatin looping. *Cell* **159**, 1665–1680 (2014).

15. G. Fudenberg, M. Imakaev, C. Lu, A. Goloborodko, N. Abdennur, L. A. Mirny, Formation of chromosomal domains by loop extrusion. *Cell Rep.* **15**, 2038–2049 (2016).

16. A. L. Sanborn, S. S. Rao, S. C. Huang, N. C. Durand, M. H. Huntley, A. I. Jewett, I. D. Bochkov, D. Chinnappan, A. Cutkosky, J. Li, K. P. Geeting, A. Gnirke, A. Melnikov, D.

McKenna, E. K. Stamenova, E. S. Lander, E. L. Aiden, Chromatin extrusion explains key features of loop and domain formation in wild-type and engineered genomes. *Proc. Natl. Acad. Sci. U.S.A.* **112**, E6456–6465 (2015).

17. E. P. Nora, A. Goloborodko, A. L. Valton, J. H. Gibcus, A. Uebersohn, N. Abdennur, J. Dekker, L. A. Mirny, B. G. Bruneau, Targeted degradation of CTCF decouples local insulation of chromosome domains from genomic compartmentalization. *Cell* **169**, 930–944.e22 (2017).

18. C. L. Wike, Y. Guo, M. Tan, R. Nakamura, D. K. Shaw, N. Diaz, A. F. Whittaker-Tademy, N. C. Durand, E. L. Aiden, J. M. Vaquerizas, D. Grunwald, H. Takeda, B. R. Cairns, Chromatin architecture transitions from zebrafish sperm through early embryogenesis. *Genome Res.* **31**, 981–994 (2021).

19. Z. Du, H. Zheng, B. Huang, R. Ma, J. Wu, X. Zhang, J. He, Y. Xiang, Q. Wang, Y. Li, J. Ma, X. Zhang, K. Zhang, Y. Wang, M. Q. Zhang, J. Gao, J. R. Dixon, X. Wang, J. Zeng, W. Xie, Allelic reprogramming of 3D chromatin architecture during early mammalian development. *Nature* **547**, 232–235 (2017).

20. Y. Ke, Y. Xu, X. Chen, S. Feng, Z. Liu, Y. Sun, X. Yao, F. Li, W. Zhu, L. Gao, H. Chen, Z. Du, W. Xie, X. Xu, X. Huang, J. Liu, 3D chromatin structures of mature gametes and structural reprogramming during mammalian embryogenesis. *Cell* **170**, 367–381.e20 (2017).

21. C. B. Hug, A. G. Grimaldi, K. Kruse, J. M. Vaquerizas, Chromatin architecture emerges during zygotic genome activation independent of transcription. *Cell* **169**, 216–228.e19 (2017).

22. I. Ozdemir, M. C. Gambetta, The role of insulation in patterning gene expression. *Genes (Basel)* **10**, 767 (2019).

23. F. Ramirez, V. Bhardwaj, L. Arrigoni, K. C. Lam, B. A. Gruning, J. Villaveces, B. Habermann, A. Akhtar, T. Manke, High-resolution TADs reveal DNA sequences underlying genome organization in flies. *Nat. Commun.* **9**, 189 (2018).

24. K. Van Bortle, M. H. Nichols, L. Li, C. T. Ong, N. Takenaka, Z. S. Qin, V. G. Corces, Insulator function and topological domain border strength scale with architectural protein occupancy. *Genome Biol.* **15**, R82 (2014).

25. K. Van Bortle, E. Ramos, N. Takenaka, J. Yang, J. E. Wahi, V. G. Corces, Drosophila CTCF tandemly aligns with other insulator proteins at the borders of H3K27me3 domains. *Genome Res.* **22**, 2176–2187 (2012).

26. Q. Wang, Q. Sun, D. M. Czajkowsky, Z. Shao, Sub-kb Hi-C in D. melanogaster reveals conserved characteristics of TADs between insect and mammalian cells. *Nat. Commun.* **9**, 188 (2018).

27. K. T. Chathoth, N. R. Zabet, Chromatin architecture reorganization during neuronal cell differentiation in Drosophila genome. *Genome Res.* **29**, 613–625 (2019).

28. A. Kaushal, J. Dorier, B. Wang, G. Mohana, M. Taschner, P. Cousin, P. Waridel, C. Iseli, A. Semenova, S. Restrepo, N. Guex, E. L. Aiden, M. C. Gambetta, Essential role of Cp190 in physical and regulatory boundary formation. *Sci. Adv.* **8**, eabl8834 (2022).

29. A. Kaushal, G. Mohana, J. Dorier, I. Ozdemir, A. Omer, P. Cousin, A. Semenova, M. Taschner, O. Dergai, F. Marzetta, C. Iseli, Y. Eliaz, D. Weisz, M. S. Shamim, N. Guex, E. Lieberman Aiden, M. C. Gambetta, CTCF loss has limited effects on global genome architecture in Drosophila despite critical regulatory functions. *Nat. Commun.* **12**, 1011 (2021).

30. N. Jiang, E. Emberly, O. Cuvier, C. M. Hart, Genome-wide mapping of boundary element-associated factor (BEAF) binding sites in Drosophila melanogaster links BEAF to transcription. *Mol. Cell. Biol.* **29**, 3556–3568 (2009).

31. M. Bartkuhn, T. Straub, M. Herold, M. Herrmann, C. Rathke, H. Saumweber, G. D. Gilfillan, P. B. Becker, R. Renkawitz, Active promoters and insulators are marked by the centrosomal protein 190. *EMBO J.* **28**, 877–888 (2009).

32. J. Li, D. S. Gilmour, Distinct mechanisms of transcriptional pausing orchestrated by GAGA factor and M1BP, a novel transcription factor. *EMBO J.* **32**, 1829–1841 (2013).

33. A. A. Soshnev, R. M. Baxley, J. R. Manak, K. Tan, P. K. Geyer, The insulator protein suppressor of Hairy-wing is an essential transcriptional repressor in the Drosophila ovary. *Development* **140**, 3613–3623 (2013).

34. M. J. Rowley, M. H. Nichols, X. Lyu, M. Ando-Kuri, I. S. M. Rivera, K. Hermetz, P. Wang, Y. Ruan, V. G. Corces, Evolutionarily conserved principles predict 3D chromatin organization. *Mol. Cell* **67**, 837–852.e7 (2017).

35. S. El-Sharnouby, B. Fischer, J. P. Magbanua, B. Umans, R. Flower, S. W. Choo, S. Russell, R. White, Regions of very low H3K27me3 partition the Drosophila genome into topological domains. *PLOS ONE* **12**, e0172725 (2017).

36. N. E. Matthews, R. White, Chromatin architecture in the fly: Living without CTCF/cohesin loop extrusion?: Alternating chromatin states provide a basis for domain architecture in Drosophila. *Bioessays* **41**, e1900048 (2019).

37. S. V. Ulianov, E. E. Khrameeva, A. A. Gavrilov, I. M. Flyamer, P. Kos, E. A. Mikhaleva, A. A. Penin, M. D. Logacheva, M. V. Imakaev, A. Chertovich, M. S. Gelfand, Y. Y. Shevelyov, S. V. Razin, Active chromatin and transcription play a key role in chromosome partitioning into topologically associating domains. *Genome Res.* **26**, 70–84 (2016).

38. M. C. Gambetta, E. E. M. Furlong, The insulator protein CTCF is required for correct hox gene expression, but not for embryonic development in Drosophila. *Genetics* **210**, 129–136 (2018).

39. K. T. Chathoth, L. A. Mikheeva, G. Crevel, J. C. Wolfe, I. Hunter, S. Beckett-Doyle, S. Cotterill, H. Dai, A. Harrison, N. R. Zabet, The role of insulators and transcription in 3D chromatin organization of flies. *Genome Res.* **32**, 682–698 (2022).

40. Y. Ogiyama, B. Schuettengruber, G. L. Papadopoulos, J. M. Chang, G. Cavalli, Polycomb-dependent chromatin looping contributes to gene silencing during drosophila development. *Mol. Cell* **71**, 73–88.e5 (2018).

41. S. M. Espinola, M. Gotz, M. Bellec, O. Messina, J. B. Fiche, C. Houbron, M. Dejean, I. Reim, A. M. Cardozo Gizzi, M. Lagha, M. Nollmann, Cis-regulatory chromatin loops arise before TADs and gene activation, and are independent of cell fate during early Drosophila development. *Nat. Genet.* **53**, 477–486 (2021).

42. A. M. Cardozo Gizzi, D. I. Cattoni, J. B. Fiche, S. M. Espinola, J. Gurgo, O. Messina, C. Houbron, Y. Ogiyama, G. L. Papadopoulos, G. Cavalli, M. Lagha, M. Nollmann, Microscopy-based chromosome conformation capture enables simultaneous visualization of genome organization and transcription in intact organisms. *Mol. Cell* **74**, 212–222.e5 (2019).

43. N. Negre, C. D. Brown, P. K. Shah, P. Kheradpour, C. A. Morrison, J. G. Henikoff, X. Feng, K. Ahmad, S. Russell, R. A. White, L. Stein, S. Henikoff, M. Kellis, K. P. White, A comprehensive map of insulator elements for the Drosophila genome. *PLOS Genet.* **6**, e1000814 (2010).

44. J. Yang, E. Sung, P. G. Donlin-Asp, V. G. Corces, A subset of Drosophila Myc sites remain associated with mitotic chromosomes colocalized with insulator proteins. *Nat. Commun.* **4**, 1464 (2013).

45. P. J. Batut, X. Y. Bing, Z. Sisco, J. Raimundo, M. Levo, M. S. Levine, Genome organization controls transcriptional dynamics during development. *Science* **375**, 566–570 (2022).

46. M. Mohan, M. Bartkuhn, M. Herold, A. Philippen, N. Heinl, I. Bardenhagen, J. Leers, R. A. White, R. Renkawitz-Pohl, H. Saumweber, R. Renkawitz, The Drosophila insulator proteins CTCF and CP190 link enhancer blocking to body patterning. *EMBO J.* **26**, 4203–4214 (2007).

47. C. Y. Pai, E. P. Lei, D. Ghosh, V. G. Corces, The centrosomal protein CP190 is a component of the gypsy chromatin insulator. *Mol. Cell* **16**, 737–748 (2004).

48. J. Vogelmann, A. Le Gall, S. Dejardin, F. Allemand, A. Gamot, G. Labesse, O. Cuvier, N. Negre, M. Cohen-Gonsaud, E. Margeat, M. Nollmann, Chromatin insulator factors involved in long-range DNA interactions and their role in the folding of the Drosophila genome. *PLOS Genet.* **10**, e1004544 (2014).

49. O. Maksimenko, M. Bartkuhn, V. Stakhov, M. Herold, N. Zolotarev, T. Jox, M. K. Buxa, R. Kirsch, A. Bonchuk, A. Fedotova, O. Kyrchanova, R. Renkawitz, P. Georgiev, Two new insulator proteins, Pita and ZIPIC, target CP190 to chromatin. *Genome Res.* **25**, 89–99 (2015).

50. S. Cuartero, U. Fresan, O. Reina, E. Planet, M. L. Espinas, Ibf1 and Ibf2 are novel CP190-interacting proteins required for insulator function. *EMBO J.* **33**, 637–647 (2014).

51. N. Zolotarev, A. Fedotova, O. Kyrchanova, A. Bonchuk, A. A. Penin, A. S. Lando, I. A. Eliseeva, I. V. Kulakovskiy, O. Maksimenko, P. Georgiev, Architectural proteins Pita, Zw5,and ZIPIC contain homodimerization domain and support specific long-range interactions in Drosophila. *Nucleic Acids Res.* **44**, 7228–7241 (2016).

52. E. Rodriguez-Carballo, L. Lopez-Delisle, Y. Zhan, P. J. Fabre, L. Beccari, I. El-Idrissi, T. H. N. Huynh, H. Ozadam, J. Dekker, D. Duboule, The HoxD cluster is a dynamic and resilient TAD boundary controlling the segregation of antagonistic regulatory landscapes. *Genes Dev.* **31**, 2264–2281 (2017).

53. R. D. Butcher, S. Chodagam, R. Basto, J. G. Wakefield, D. S. Henderson, J. W. Raff, W. G. Whitfield, The Drosophila centrosome-associated protein CP190 is essential for viability but not for cell division. *J. Cell Sci.* **117**, 1191–1199 (2004).

54. W. S. Klug, D. Bodenstein, R. C. King, Oogenesis in the suppressor of hairy-wing mutant of Drosophila melanogaster. I. Phenotypic characterization and transplantation experiments. *J. Exp. Zool.* **167**, 151–156 (1968).

55. S. Roy, M. K. Gilbert, C. M. Hart, Characterization of BEAF mutations isolated by homologous recombination in Drosophila. *Genetics* **176**, 801–813 (2007).

56. M. V. Staller, D. Yan, S. Randklev, M. D. Bragdon, Z. B. Wunderlich, R. Tao, L. A. Perkins, A. H. Depace, N. Perrimon, Depleting gene activities in early Drosophila embryos with the "maternal-Gal4-shRNA" system. *Genetics* **193**, 51–61 (2013).

57. J. Zuin, J. R. Dixon, M. I. van der Reijden, Z. Ye, P. Kolovos, R. W. Brouwer, M. P. van de Corput, H. J. van de Werken, T. A. Knoch, I. W. F. van IJcken, F. G. Grosveld, B. Ren, K. S.

Wendt, Cohesin and CTCF differentially affect chromatin architecture and gene expression in human cells. *Proc. Natl. Acad. Sci. U.S.A.* **111**, 996–1001 (2014).

58. C. Matthey-Doret, L. Baudry, A. Breuer, R. Montagne, N. Guiglielmoni, V. Scolari, E. Jean, A. Campeas, P. H. Chanut, E. Oriol, A. Meot, L. Politis, A. Vigouroux, P. Moreau, R. Koszul, A. Cournac, Computer vision for pattern detection in chromosome contact maps. *Nat. Commun.* **11**, 5795 (2020).

59. S. De Renzis, O. Elemento, S. Tavazoie, E. F. Wieschaus, Unmasking activation of the zygotic genome using chromosomal deletions in the Drosophila embryo. *PLoS Biol.* **5**, e117 (2007).

60. S. E. Celniker, S. Sharma, D. J. Keelan, E. B. Lewis, The molecular genetics of the bithorax complex of Drosophila: Cis-regulation in the abdominal-B domain. *EMBO J.* **9**, 4277–4286 (1990).

61. M. J. Pankratz, E. Seifert, N. Gerwin, B. Billi, U. Nauber, H. Jackle, Gradients of Krüppel and knirps gene products direct pair-rule gene stripe patterning in the posterior region of the Drosophila embryo. *Cell* **61**, 309–317 (1990).

62. S. Small, A. Blair, M. Levine, Regulation of even-skipped stripe 2 in the Drosophila embryo. *EMBO J.* **11**, 4047–4057 (1992).

63. L. Braccioli, E. de Wit, CTCF: A Swiss-army knife for genome organization and transcription regulation. *Essays Biochem.* **63**, 157–165 (2019).

64. N. Kubo, H. Ishii, X. Xiong, S. Bianco, F. Meitinger, R. Hu, J. D. Hocker, M. Conte, D. Gorkin, M. Yu, B. Li, J. R. Dixon, M. Hu, M. Nicodemi, H. Zhao, B. Ren, Promoter-proximal CTCF binding promotes distal enhancer-dependent gene activation. *Nat. Struct. Mol. Biol.* **28**, 152–161 (2021).

65. G. N. Filippova, S. Fagerlie, E. M. Klenova, C. Myers, Y. Dehner, G. Goodwin, P. E. Neiman, S. J. Collins, V. V. Lobanenkov, An exceptionally conserved transcriptional

repressor, CTCF, employs different combinations of zinc fingers to bind diverged promoter sequences of avian and mammalian c-myc oncogenes. *Mol. Cell. Biol.* **16**, 2802–2813 (1996).

66. R. Pena-Hernandez, M. Marques, K. Hilmi, T. Zhao, A. Saad, M. A. Alaoui-Jamali, S. V. del Rincon, T. Ashworth, A. L. Roy, B. M. Emerson, M. Witcher, Genome-wide targeting of the epigenetic regulatory protein CTCF to gene promoters by the transcription factor TFII-I. *Proc. Natl. Acad. Sci. U.S.A.* **112**, E677–E686 (2015).

67. L. L. P. Hanssen, M. T. Kassouf, A. M. Oudelaar, D. Biggs, C. Preece, D. J. Downes, M. Gosden, J. A. Sharpe, J. A. Sloane-Stanley, J. R. Hughes, B. Davies, D. R. Higgs, Tissue-specific CTCF-cohesin-mediated chromatin architecture delimits enhancer interactions and function in vivo. *Nat. Cell Biol.* **19**, 952–961 (2017).

68. M. Fujioka, G. Sun, J. B. Jaynes, The Drosophila eve insulator Homie promotes eve expression and protects the adjacent gene from repression by polycomb spreading. *PLOS Genet.* **9**, e1003883 (2013).

69. J. Serano, G. M. Rubin, The Drosophila synaptotagmin-like protein bitesize is required for growth and has mRNA localization sequences within its open reading frame. *Proc. Natl. Acad. Sci. U.S.A.* **100**, 13368–13373 (2003).

70. H. L. Liang, C. Y. Nien, H. Y. Liu, M. M. Metzstein, N. Kirov, C. Rushlow, The zinc-finger protein Zelda is a key activator of the early zygotic genome in Drosophila. *Nature* **456**, 400–403 (2008).

71. C. Anania, R. D. Acemel, J. Jedamzick, A. Bolondi, G. Cova, N. Brieske, R. Kuhn, L. Wittler, F. M. Real, D. G. Lupianez, In vivo dissection of a clustered-CTCF domain boundary reveals developmental principles of regulatory insulation. *Nat. Genet.* **54**, 1026–1036 (2022).

72. L. Li, X. Lyu, C. Hou, N. Takenaka, H. Q. Nguyen, C. T. Ong, C. Cubenas-Potts, M. Hu, E. P. Lei, G. Bosco, Z. S. Qin, V. G. Corces, Widespread rearrangement of 3D chromatin organization underlies polycomb-mediated stress-induced silencing. *Mol. Cell* **58**, 216–231 (2015).

73. I. Bag, S. Chen, L. F. Rosin, Y. Chen, C. Y. Liu, G. Y. Yu, E. P. Lei, M1BP cooperates with CP190 to activate transcription at TAD borders and promote chromatin insulator activity. *Nat. Commun.* **12**, 4170 (2021).

74. C. C. Galouzis, E. E. M. Furlong, Regulating specificity in enhancer-promoter communication. *Curr. Opin. Cell Biol.* **75**, 102065 (2022).

75. V. S. Chopra, J. Cande, J. W. Hong, M. Levine, Stalled Hox promoters as chromosomal boundaries. *Genes Dev.* **23**, 1505–1509 (2009).

76. S. J. Gratz, F. P. Ukken, C. D. Rubinstein, G. Thiede, L. K. Donohue, A. M. Cummings, K. M. O'Connor-Giles, Highly specific and efficient CRISPR/Cas9-catalyzed homology-directed repair in Drosophila. *Genetics* **196**, 961–971 (2014).

77. J. A. Campos-Ortega, V. Hartenstein, *The embryonic development of Drosophila melanogaster*. (Springer, 2013).

78. S. A. Blythe, E. F. Wieschaus, Zygotic genome activation triggers the DNA replication checkpoint at the midblastula transition. *Cell* **160**, 1169–1181 (2015).

79. S. Bonn, R. P. Zinzen, A. Perez-Gonzalez, A. Riddell, A. C. Gavin, E. E. Furlong, Cell type-specific chromatin immunoprecipitation from multicellular complex samples using BiTS-ChIP. *Nat. Protoc.* **7**, 978–994 (2012).

80. H. S. Kaya-Okur, D. H. Janssens, J. G. Henikoff, K. Ahmad, S. Henikoff, Efficient low-cost chromatin profiling with CUT&Tag. *Nat. Protoc.* **15**, 3264–3283 (2020).

81. M. M. Harrison, X. Y. Li, T. Kaplan, M. R. Botchan, M. B. Eisen, Zelda binding in the early Drosophila melanogaster embryo marks regions subsequently activated at the maternal-to-zygotic transition. *PLOS Genet.* **7**, e1002266 (2011).

82. J. Schindelin, I. Arganda-Carreras, E. Frise, V. Kaynig, M. Longair, T. Pietzsch, S. Preibisch, C. Rueden, S. Saalfeld, B. Schmid, J. Y. Tinevez, D. J. White, V. Hartenstein, K. Eliceiri, P.

Tomancak, A. Cardona, Fiji: An open-source platform for biological-image analysis. *Nat. Methods* **9**, 676–682 (2012).

83. I. E. Schor, G. Bussotti, M. Males, M. Forneris, R. R. Viales, A. J. Enright, E. E. M. Furlong, Non-coding RNA expression, function, and variation during Drosophila embryogenesis. *Curr. Biol.* **28**, 3547–3561.e9 (2018).

84. M. Martin, *Cutadapt removes adapter sequences from high-throughput sequencing reads*. *EMBnet J.* **17**, 1–10 (2011).

85. A. Dobin, C. A. Davis, F. Schlesinger, J. Drenkow, C. Zaleski, S. Jha, P. Batut, M. Chaisson, T. R. Gingeras, STAR: Ultrafast universal RNA-seq aligner. *Bioinformatics* **29**, 15–21 (2013).

86. B. Li, C. N. Dewey, RSEM: Accurate transcript quantification from RNA-seq data with or without a reference genome. *BMC Bioinformatics* **12**, 323 (2011).

87. M. I. Love, W. Huber, S. Anders, Moderated estimation of fold change and dispersion for RNA-seq data with DESeq2. *Genome Biol.* **15**, 550 (2014).

88. V. Ramani, X. Deng, R. Qiu, K. L. Gunderson, F. J. Steemers, C. M. Disteche, W. S. Noble, Z. Duan, J. Shendure, Massively multiplex single-cell Hi-C. *Nat. Methods* **14**, 263–266 (2017).
